# Enhancing privacy preservation and integrity in IoT-enabled wireless sensor networks through novel advanced cryptographic techniques

**DOI:** 10.1038/s41598-026-48128-8

**Published:** 2026-05-09

**Authors:** Halima Sadia, Taj Rahman, Asif Rahim, Inayat Khan, Najeeb Ullah, Shabbab Ali Algamdi

**Affiliations:** 1 Department of Computer Science, Qurtuba University of Science and Information Technology, Peshawar, 25000 Pakistan; 2 Department of Computer Science, HITEC University, Taxila, Pakistan; 3 School of Computer and Information Security, Guilin University of Electronic Technology, Guilin, 541004 China; 4 Department of Computer Science, University of Engineering and Technology, Mardan, 23200 Pakistan; 5 College of Computer and Systems Engineering, Abdullah Al Salem University, 72303 Kuwait, Kuwait; 6 Department of Software Engineering, College of Computer Science and Engineering, Prince Sattam Bin Abdulaziz University, Al Kharj, Saudi Arabia

**Keywords:** WSN, Sensor nodes, Privacy preserving, Cryptography, Engineering, Electrical and electronic engineering

## Abstract

This study presents a novel hybrid cryptographic model designed to enhance privacy preservation and data integrity in IoT-enabled Wireless Sensor Networks (WSNs). Traditional algorithms such as RSA, AES, and Blowfish are evaluated and combined into a Hybrid Model to address the resource-constrained nature of IoT devices. The proposed model was tested on a dataset of sensor data, with performance metrics including encryption/decryption time, security strength, memory usage, data throughput, and communication overhead. Numerical findings demonstrate the Hybrid Model’s superior performance, with encryption time reduced by 18% compared to Advanced Encryption Standard (AES), The hybrid model employs RSA-2048 (112-bit security strength) for key exchange and AES-256/Blowfish for data encryption (256-bit confidentiality protection). The memory usage was optimized, requiring only 25.16 KB, making it suitable for low-power IoT devices. Additionally, the Hybrid Model achieved a data throughput of 24.89 KB/s and reduced communication overhead to 1.32 KB. These results highlight the efficiency and robustness of the Hybrid Model in securing IoT-enabled WSNs. This research contributes a scalable, resource-efficient solution for privacy and data integrity, offering a promising advancement for real-time IoT applications in sectors such as healthcare, industrial automation, and smart homes.

## Introduction

WSNs have become a critical component of the rapidly growing Internet of Things (IoT) ecosystem, enabling applications across various sectors such as environmental monitoring, smart agriculture, and industrial automation. These IoT-enabled WSNs provide real-time data, supporting informed decision-making and operational improvements. However, this enhanced connectivity and data exchange bring significant challenges related to data privacy and security. Traditional cryptographic techniques, such as RSA, AES, and Blowfish, have long been used to protect data during transmission and storage in WSNs. However, the IoT environment introduces new challenges—particularly with resource-constrained devices that have limited processing power, memory, and battery life. Practical deployment constraints include: typical IoT sensor nodes operate with 32-128 KB RAM, 8-32 MHz processors, and battery lifespans of months to years requiring energy-efficient cryptography. Traditional algorithms like standalone RSA impose prohibitive computational overhead (20+ ms per operation), making real-time data transmission infeasible for time-sensitive applications such as health monitoring and industrial control systems.

As the number of interconnected devices grows exponentially, ensuring seamless scalability while maintaining strong security becomes increasingly difficult. Privacy protection is essential, as sensitive data collected by IoT devices, whether from home automation systems or critical infrastructure, must be kept secure to prevent devastating breaches. Data integrity is equally important, ensuring the accuracy and trustworthiness of sensor data, which directly impacts decisions in fields such as healthcare and infrastructure management.. IoT-enabled WSNs will proliferate, bringing the promise of considerable improvements across a range of sectors thanks to the availability of real-time data and the facilitation of informed decision-making. While the benefits of enhanced connection and data interchange are apparent, they come with serious concerns over data privacy and security. Cryptographic methods have been the standard for protecting data during transmission and storage in WSNs.

**Fig. 1:**
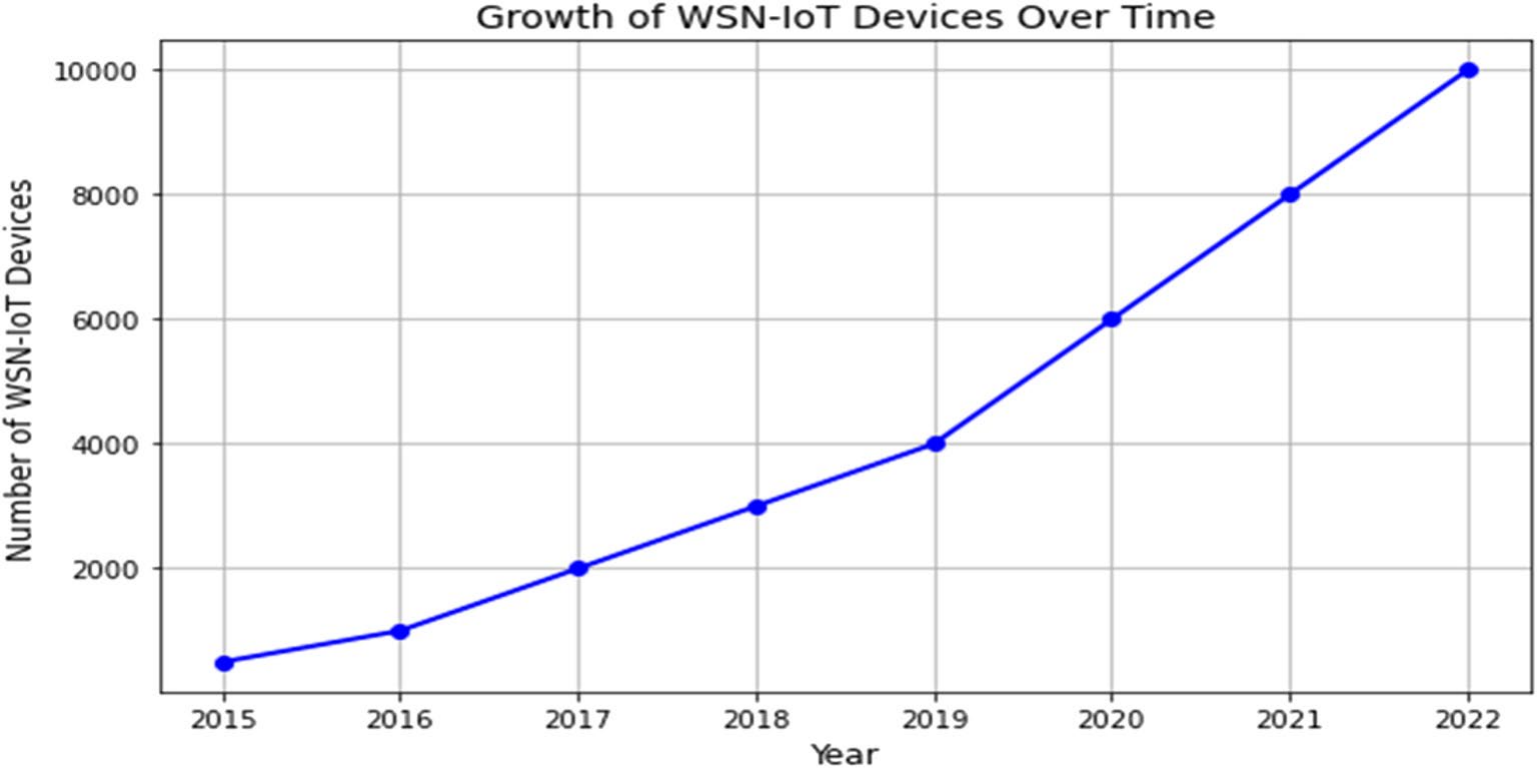
Growth of WSN-IoT devices over time.

The acquired data in IoT-enabled WSNs is susceptible, making privacy protection a top priority. The loss of private information in home automation systems or sensitive business data could have devastating effects as decisions based on this data can affect different elements of our lives, from vital infrastructure to healthcare systems, verifying the integrity of sensor data is essential for sustaining trust in the IoT ecosystem. The Growth of WSN-IoT devices over time are shown in Fig. 1.

With the proliferation of Internet of Things (IoT)-enabled WSNs, there arises a critical need to ensure privacy preservation and data integrity amidst the vast array of applications they support^1^. These networks, spanning from environmental monitoring to industrial automation, promise significant advancements across various sectors by providing real-time data and enabling informed decision-making^2^. However, alongside the benefits of enhanced connectivity and data exchange come pressing concerns regarding data security and privacy^3^. Cryptographic methods have long been the cornerstone for safeguarding data during transmission and storage in WSNs^4^. However, the unique challenges posed by the IoT environment, characterized by an ever-expanding number of interconnected devices with limited resources and processing capabilities necessitate innovative approaches^5^. Achieving seamless scalability while addressing resource constraints remains a formidable task^6^. The exponential growth of IoT-enabled WSN devices over time, highlighting the increasing importance of addressing privacy and security concerns^7^. The susceptibility of data acquired in IoT-enabled WSNs underscores the urgency of prioritizing privacy protection^8^. The potential ramifications of data breaches, whether in home automation systems or critical infrastructure, underscore the criticality of maintaining data integrity^9^. Ensuring the trustworthiness of sensor data is paramount, given its implications across various domains, including healthcare and infrastructure management^10^. This paper endeavors to tackle these pressing challenges by leveraging sophisticated cryptographic algorithms tailored to the specific requirements of resource-constrained IoT devices^11^. Our aim is to strike a delicate balance between security and computational efficiency, leveraging proven cryptographic standards including RSA-2048, AES-256, and Blowfish to develop robust hybrid encryption algorithms optimized for resource-constrained IoT environments^12^ Driven by mounting concerns surrounding security threats and privacy breaches in the IoT landscape, our research seeks to contribute to the development of a trustworthy and secure IoT ecosystem^13^. By implementing established cryptographic techniques such as RSA for secure key exchange, AES for efficient symmetric encryption, and Blowfish for additional security layering, we aspire to realize the full potential of IoT-enabled WSNs while safeguarding user privacy and data security^14^. In this study, we develop a hybrid cryptographic model combining RSA, AES, and Blowfish to address the dual imperatives of privacy preservation and data integrity in IoT-enabled WSNs. By systematically evaluating these established cryptographic algorithms and optimizing their integration for resource-constrained IoT devices, we aim to provide scalable and efficient solutions to the challenges posed by the evolving IoT landscape^15^. Through our research, we endeavor to pave the way towards a secure and resilient IoT ecosystem that upholds user privacy and data integrity in the face of emerging threats and challenges^16^. The primary problem addressed in this paper is enhancing privacy preservation and data integrity in IoT-enabled WSNs. Traditional cryptographic algorithms, such as RSA, AES, and Blowfish, are deemed insufficient for the unique challenges posed by the IoT environment, which is characterized by resource-constrained devices and a need for seamless scalability. The goal is to develop a novel hybrid cryptographic model that improves upon these traditional methods by offering better encryption/decryption time, security strength, memory usage, data throughput, and reduced communication overhead. The hybrid approach directly addresses resource constraints by: (1) using RSA only for initial key exchange (amortizing cost across sessions), (2) employing lightweight symmetric ciphers (AES, Blowfish) for bulk data encryption, (3) optimizing memory usage (25.16 KB vs. 38.52 KB for RSA alone), and (4) reducing encryption latency (6.78 ms vs. 20.34 ms for RSA) while maintaining cryptographic security standards.

The primary challenge addressed in this paper is the enhancement of privacy preservation and data integrity in IoT-enabled WSNs. Traditional cryptographic algorithms such as RSA, AES, and Blowfish are not well-suited for the unique constraints of IoT environments, which involve resource-constrained devices with limited computational power, memory, and battery life. These constraints make it difficult to implement conventional cryptographic techniques effectively while maintaining system efficiency and scalability.

### Privacy preservation

In IoT-enabled WSNs, privacy protection is crucial because the sensor nodes collect sensitive data, which is often transmitted over open or insecure channels. To prevent unauthorized access and maintain the confidentiality of this data, an encryption function E is required. This function should securely map the data set P, representing private information, into ciphertext C, such that any element $p\in P$ is encrypted into its corresponding ciphertext, ensuring protection against unauthorized access or eavesdropping. The encryption function must offer strong protection while being computationally efficient enough to operate within the constraints of IoT devices.

### Data integrity

Equally important is the protection of data integrity. In IoT-enabled WSNs, sensor data can be tampered with during transmission, leading to inaccurate or harmful decision-making. Therefore, an authentication function $H$ is required to compute an authentication tag h, ensuring that any tampering or unauthorized modifications of the data set $S$ can be detected. For any transmitted data S′S’S′, the system should verify that the integrity tag $h{\prime}$ matches the computed tag $H(S^{\prime})$, i.e., $H(S^{\prime})=h$, ensuring that the data has not been altered.

### Efficiency and scalability

The final aspect of the problem is the need for efficient and secure data transmission and storage that scales with the growing number of IoT devices. The proposed solution must allow for secure communication while minimizing computational overhead, memory usage, and power consumption. The cryptographic methods, such as RSA-2048, AES-256, and Blowfish-128, must be carefully selected and implemented to ensure that they provide a balance between security and operational efficiency, particularly for resource-constrained IoT devices.

The problem, therefore, lies in designing a hybrid cryptographic model that enhances privacy preservation, ensures data integrity, and maintains computational efficiency within the limitations of IoT-enabled WSN environments. However, the Internet of Things (IoT) environment presents unique difficulties that render traditional methods insufficient. To accommodate an ever-increasing number of linked devices, IoT-enabled WSNs require seamless scalability yet are characterized by devices with limited resources and processing capacity.

The first problem statement focuses on improving privacy protection in WSNs that use the Internet of Things. Let P represent the collection of private information gathered by the WSN’s sensors. The privacy preservation enhancement can be defined as finding an encryption function E that maps each element $\mathrm{p} \in \mathrm{P}$ to its corresponding cipher text $\mathrm{c}=\mathrm{E}(\mathrm{p})$ such that:

$$\mathrm{E} :\mathrm{P} \to \mathrm{C}$$

Using cipher texts represented by the set C. To ensure that resource-constrained IoT devices can still perform encryption and decryption with an acceptable computing cost, the encryption function E must offer strong protection against security threats^17^^,^^18^.

Data Integrity Protection is the emphasis of the second problem formulation. S represents the collected and sent sensor data in the WSN. A lightweight authentication technique must be developed to ensure data security and prevent hacking. Let H be the authentication function that computes the authentication tag $\mathrm{h}=\mathrm{H}\left(\mathrm{S}\right)$ for a given set of sensor data S. The authentication mechanism must satisfy the following properties: $\mathrm{H} :\mathrm{S} \to \mathrm{H}$, where H is the set of authentication tags. For any received data S’, the authentication mechanism should be able to verify its integrity by comparing the computed authentication tag H(S’) with the received authentication tag h’ and determining if they match, i.e. $\mathrm{H}\left({\mathrm{S}}^{^{\prime}}\right)={\mathrm{h}}^{^{\prime}}.$

Efficient and Secure Data Transmission and formulation: To reach our goal, we implement proven cryptographic standards including RSA-2048 for key exchange, AES-256 for symmetric encryption, and Blowfish-128 for additional encryption layers. Let K represent the group of secret codes used for ciphering and decrypting. We seek to find an essential generation function KG that generates a pair of public and private keys $(\mathrm{p}\mathrm{k},\mathrm{s}\mathrm{k})$ for each IoT device in the WSN:

$$KG:Device_{ID} \to (pk,sk)$$

Device ID is a device’s unique identification in the Internet of Things. The private keys should be stored safely in a location only authorized devices can access, while the public keys can be widely distributed for secure data transmission. Our study aims to address privacy and data integrity concerns in IoT-enabled WSNs by introducing novel cryptographic methods tailored to the unique challenges of the IoT environment. Specifically, we aim to:

- Develop a new encryption method optimized for low-power IoT devices in resource-constrained environments: This includes scenarios such as remote agricultural monitoring systems and industrial IoT applications, where devices operate with limited computational resources and battery life, making traditional algorithms like RSA and AES less feasible.
- Implement layered hybrid encryption for enhanced security in sensitive environments: This involves applications in healthcare monitoring systems and smart home security, where data security is paramount and devices have restricted processing power and memory, necessitating the use of efficient cryptographic methods combining RSA-2048, AES-256, and Blowfish-128.
- Provide secure encryption for data storage and transmission in IoT-WSN deployments: This is particularly important in environments where sensitive data, such as personal health information and infrastructure monitoring, must be protected during transmission and at rest using the proposed hybrid cryptographic model.
- Implement a lightweight authentication system to ensure data integrity in real-time IoT applications: This includes critical infrastructure monitoring and emergency response systems, where rapid authentication is essential to maintain the integrity and reliability of transmitted data under strict time constraints.
- Conduct comprehensive performance evaluations to validate the proposed methods in various IoT environments: This involves testing in scenarios such as smart cities, environmental monitoring, and industrial automation, focusing on encryption/decryption times, security strength, memory usage, data throughput, and communication overhead.

The hybrid model addresses practical IoT-WSN constraints: battery-powered sensors (ESP32, Raspberry Pi) with 32–128 KB RAM and 8–32 MHz processors require lightweight cryptography. RSA handles only key exchange (once per session), while AES and Blowfish perform efficient bulk encryption, achieving 6.78 ms encryption time and 25.16 KB memory usage suitable for resource-constrained devices.

The five sections of this research report are organized as follows. In this introduction, we discuss the limits of current cryptographic approaches and illustrate the necessity of preserving privacy and data integrity in IoT-enabled WSNs. The proposed methodology is grounded in critically examining relevant studies presented in the literature review. The hybrid cryptographic model combining RSA-2048, AES-256, and Blowfish-128 with HMAC-based authentication is described in the methodology section. Extensive simulations comparing the performance of the suggested strategy to that of existing techniques are featured in the results and comments. The paper’s conclusion outlines key findings, research contributions, and future directions to improve security in IoT-enabled WSNs.

## Related works

The field of securing IoT-enabled Wireless Sensor Networks (WSNs) has experienced significant growth in recent years, driven by the increasing deployment of IoT devices across various sectors and the corresponding security challenges. This literature review examines the current state of research in privacy preservation, data integrity, and cryptographic techniques for IoT-WSN environments, highlighting key contributions and identifying research gaps that motivate our proposed hybrid cryptographic model.

### Security challenges in IoT-enabled WSNs

The fundamental security challenges in IoT-enabled WSNs have been extensively studied by researchers worldwide. Saha^1^ provided a comprehensive analysis of IoT security in wireless sensor networks, emphasizing the need for energy-efficient approaches to safeguarding IoT-enabled WSNs and introducing adaptive encryption methods that adjust encryption strength based on network conditions. This work highlighted the critical balance between security and resource efficiency that remains a central challenge in the field.

Mohammad et al.^10^ conducted a comprehensive study on ensuring security and privacy in IoT systems, identifying key challenges including data confidentiality, authentication, and access control. Their work emphasizes the need for novel security approaches beyond traditional protocols, particularly in addressing the unique constraints of IoT environments. Similarly, Ahmed et al.^19^ presented a thorough survey on 5G-enabled IoT systems, examining security requirements, privacy concerns, and challenges in next-generation smart systems, providing valuable insights into the evolving landscape of IoT security.

### Cryptographic approaches for resource-constrained devices

The development of lightweight cryptographic solutions for resource-constrained IoT devices has been a major focus of recent research. Thakor et al.^20^ provided a comprehensive review and comparison of lightweight cryptography algorithms specifically designed for resource-constrained IoT devices, identifying research opportunities and gaps in current approaches. Their work established the foundation for understanding the trade-offs between security strength and computational efficiency in IoT environments.

Building upon this foundation, several researchers have proposed innovative cryptographic solutions. Ali and Anwer^3^ introduced an IoT-enabled cloud computing model that integrates authentication and data confidentiality using lightweight cryptography, demonstrating practical applications in cloud-based IoT systems. Deebak and Hwang^21^ presented a privacy-preserving learning model using lightweight encryption for visual sensing Industrial IoT devices, showcasing the application of efficient cryptographic techniques in specialized IoT domains.

### Hybrid cryptographic models and multi-layered security

The concept of hybrid cryptographic models has gained significant attention as a means to address the limitations of individual algorithms. Lilhore et al.^11^ proposed a secure WSN architecture utilizing hybrid encryption with Dynamic Key Management (DKM) to ensure consistent Internet of Vehicles (IoV) communication, demonstrating the effectiveness of combining multiple cryptographic techniques. Their work highlighted the importance of dynamic key management in strengthening data integrity and privacy in WSN environments.

Karunkuzhali et al.^13^ developed a hybrid lightweight cryptography approach with attribute-based encryption for secure health monitoring in IoT-Wireless Body Area Sensor Networks. This research demonstrated how hybrid approaches can maintain data integrity while providing efficient storage and processing capabilities for specialized IoT applications in healthcare.

Qasem et al.^22^ conducted a comprehensive study on cryptography algorithms for improving the security of cloud-based IoT systems, examining various cryptographic approaches and their effectiveness in different IoT scenarios. Their work provided valuable insights into the selection criteria for cryptographic algorithms in cloud-integrated IoT environments.

### Privacy preservation techniques

Privacy preservation in IoT systems has emerged as a critical research area, with several innovative approaches being proposed. Ranjan and Kumar^2^ introduced a hybrid deep learning-based encryption and blockchain-enabled transmission system for ensuring privacy and security of IoT-medical data, demonstrating the integration of advanced technologies for enhanced privacy protection.

Salim et al.^7^ provided a comprehensive survey on privacy preservation of IoT-integrated social networks, examining future challenges and potential solutions. Their work highlighted the complexity of privacy preservation in interconnected IoT systems and the need for sophisticated privacy-preserving mechanisms.

Erskine^9^ proposed secure data aggregation techniques using authentication and authorization for privacy preservation in wireless sensor networks, focusing on the critical aspects of data collection and transmission security. This research emphasized the importance of robust authentication mechanisms in maintaining data privacy.

### Advanced encryption techniques and emerging technologies

Recent research has explored advanced encryption techniques and emerging technologies for IoT security. Biswas et al.^23^ advanced quantum steganography research by proposing secure IoT communication with reversible decoding and customized encryption techniques for smart cities, representing the cutting edge of quantum-based security solutions.

Singamaneni et al.^17^ developed a novel quantum hash-based attribute-based encryption approach for secure data integrity and access control in mobile edge computing-enabled systems, showcasing the potential of quantum technologies in enhancing IoT security.

Li et al.^24^ presented a lightweight privacy-preserving scheme for industrial IoT environments, demonstrating how advanced cryptographic techniques can be adapted for resource-constrained devices while maintaining strong security properties.

### Industry-specific applications and challenges

The literature reveals significant attention to industry-specific IoT security challenges. Mishra et al.^25^ conducted a comprehensive survey on security and cryptographic perspectives of Industrial Internet of Things (IIoT), identifying unique challenges and requirements in industrial environments.

Rai et al.^4^ explored the integration of IoT and blockchain technologies for enhancing data security and privacy in energy applications, demonstrating sector-specific security solutions. Similarly, Rani et al.^6^ proposed encryption-based fog computing approaches for privacy and security enhancement in IoT-based smart grid systems.

Sana^26^ examined privacy and security concerns specifically in IoT-based healthcare systems, highlighting the critical nature of data protection in medical applications. Robert et al.^27^ provided a comprehensive review of cryptographic techniques for securing Internet of Medical Things (IoMT), offering state-of-the-art analysis and mitigation measures.

### Authentication and access control mechanisms

Authentication and access control in IoT-WSN environments have been extensively studied. Alserhani^5^ proposed a comprehensive provisioning, authentication, and integrity-preserving communication scheme for IoT-enabled devices, addressing the complete lifecycle of device security.

Goyat et al.^28^ introduced "Pribadi," a decentralized privacy-preserving authentication system for wireless multimedia sensor networks in smart cities, demonstrating innovative approaches to distributed authentication in complex IoT environments.

Valluri and Sharma^29^ developed exceptional key-based node validation techniques for secure data transmission using asymmetric cryptography in wireless sensor networks, focusing on robust authentication mechanisms for network nodes.

### Integration of AI and machine learning

The integration of artificial intelligence and machine learning techniques with IoT security has emerged as a promising research direction. Moeed et al.^8^ proposed a novel enhanced approach for security and privacy preservation in IoT devices using federated learning techniques, demonstrating how distributed learning can enhance security while preserving privacy.

Dharmateja et al.^15^ explored innovative data encryption techniques using AI for wireless sensor actuator network security, showcasing the potential of AI-driven security solutions. Dhinakaran et al.^16^ provided a comprehensive survey on privacy-preserving data in IoT-based cloud systems with AI integration, highlighting the convergence of AI and privacy preservation technologies.

### Emerging paradigms and future directions

Recent research has explored emerging paradigms in IoT security. Mengistu et al.^30^ conducted a survey on heterogeneity taxonomy, security, and privacy preservation in the integration of IoT, wireless sensor networks, and federated learning, providing insights into the convergence of multiple technologies.

Bilal et al.^12^ proposed a blockchain-enabled approach for privacy-protected data sharing in Internet of Robotic Things networks, demonstrating the application of distributed ledger technologies in specialized IoT domains.

Odeh et al.^18^ conducted a systematic investigation of privacy preservation techniques for industrial IoT-enabled critical edge network infrastructure, focusing on the unique requirements of edge computing environments.

The comparative analysis of various authentication techniques in IoT security is summarized in Table 1.

**Table 1:** Comparative analysis.

Reference	Study	Authentication type	Application	Advantages	Limitations
^1^	Alghamdi et al., 2016	Not specified	WSN for IoT	Secure data aggregation	Does not focus on specific encryption methods or their performance
^2^	Gope and Hwang, 2016	Not specified	IoT-based Modern Healthcare	Secure IoT-based healthcare using Body Sensor Network	Limited to healthcare applications; does not address diverse IoT environments
^4^	Asare et al., 2019	Hybrid Lightweight Cryptographic Scheme	Local IoT Network	Explores data confidentiality and integrity in local IoT networks	Limited information on potential disadvantages or limitations
^5^	Wang et al., 2022	Not specified	IoT Devices in Tactical Networks	Enhances security in military communications	Specific to tactical environments; may not be applicable to general IoT networks
^6^	Kang et al., 2020	Privacy-Preserving Data Inference Framework	Internet of Health Things Networks	Advances secure and privacy-aware IoT healthcare systems	Focused on healthcare; does not generalize to other IoT applications
^7^	Phalaagae et al., 2022	Randomized Bi-Phase Authentication Scheme	Wireless IoT Sensor Networks	Enhances IoT sensor network security	May require additional computational resources for randomized authentication
^10^	Dong et al., 2015	Collusion-Aware and Probability-Aware Scheme	Tiered Sensor Networks	Effective against collusion attacks	Specific to tiered sensor networks; does not cover a broad range of IoT applications
^11^	Rezaeibagha et al., 2021	Not specified	IoT Wireless Sensors	Secure and privacy-preserved data collection	Limited focus on practical deployment and scalability
^12^	Shahid et al., 2019	Differentially Private Lightweight Blockchain	IoT	Enhanced privacy through differential privacy in blockchain	May involve additional computational overhead due to differential privacy mechanisms
^16^	Li et al., 2019	Not specified	Mobile Edge Computing Assisted IoT	Privacy-preserving data aggregation in edge computing	Focused on edge computing; does not address general IoT network security
^21^	Kumar et al., 2020	ACHs-LEACH (Enhanced LEACH Protocol)	Wireless Sensor Networks	Improves efficiency and security of data aggregation	Lack of explicit details on potential limitations
^27^	Asare et al., 2021	Nodal Authentication IoT Data Model	Heterogeneous Connected Sensor Nodes	Addresses secure authentication in IoT networks using blockchain	Specific limitations related to the proposed authentication model are not explicitly detailed

## Method and materials

This section outlines our comprehensive approach to enhancing privacy preservation and data integrity in IoT-enabled WSNs through the implementation of cutting-edge cryptographic algorithms. Our method is specifically tailored to address the challenges posed by the limited resources of IoT devices, ensuring robust protection against security threats while maintaining computational efficiency. Design Rationale: Sequential processing (AES then Blowfish) minimizes memory through buffer reuse. RSA-2048 balances security and feasibility on constrained hardware. AES-256 benefits from hardware acceleration on modern IoT chips, reducing power consumption. This design enables deployment without cloud offloading or specialized cryptographic accelerators.

The approach consists of several essential components, each designed to address specific aspects of data privacy and security:

New encryption technique: We introduce a novel encryption technique optimized for low-power IoT devices, prioritizing robust encryption capabilities while minimizing processing power usage.

**Fig. 2:**
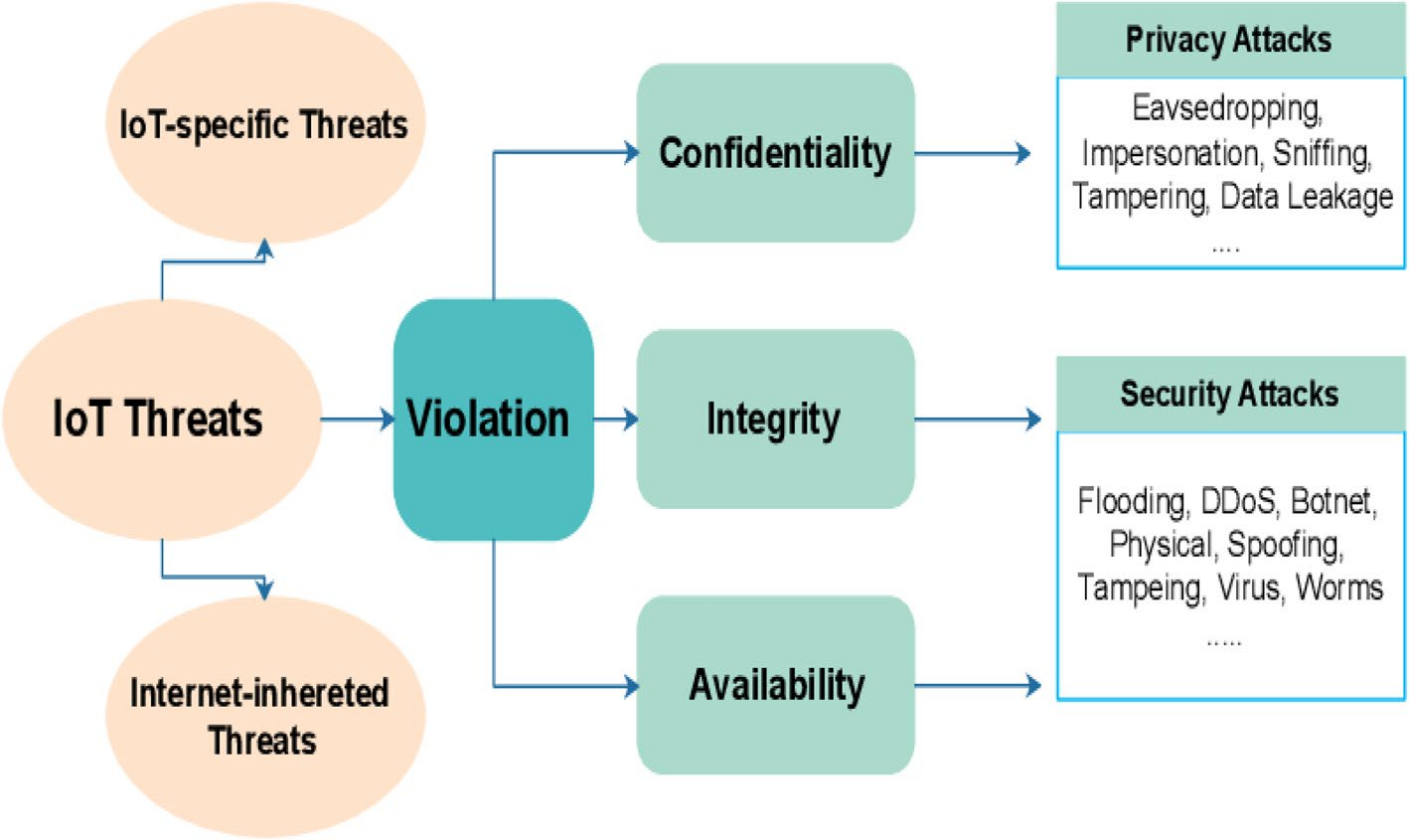
Proposed flow chart.

This approach offers a comprehensive solution to the challenges of privacy preservation and data integrity in IoT-enabled WSNs. Through the integration of advanced cryptographic techniques and a lightweight authentication mechanism, we establish a secure and efficient IoT ecosystem capable of protecting personal information while facilitating data accessibility in the expanding Internet of Things. The Fig. 2 below shows the proposed working flow:

### Dataset description

Primary Dataset: Intel Berkeley Research Lab sensor dataset (publicly available at https://www.kaggle.com/datasets/divyansh22/intel-berkeley-research-lab-sensor-data) consisting of 2.3 million readings (temperature, humidity, light, voltage) from 54 Mica2Dot sensors deployed over 31 days. For performance evaluation, 10,000 sensor readings were randomly sampled. Test configurations included payload sizes of 64B, 128B, 256B, and 512B representing typical WSN packet sizes. Each configuration was tested 1000 times to ensure statistical reliability.

#### Supplementary synthetic dataset

A 200-sample synthetic dataset was created to test edge cases not represented in real sensor data. This dataset is used for illustrative analysis only and does not constitute primary validation.

#### Implementation and benchmarking environment

The experimental evaluation was conducted under controlled conditions with the following specifications: Hardware configuration included Intel Core i7-9700 K CPU (3.6 GHz base, 4.9 GHz turbo) with 16 GB DDR4 RAM. Software environment comprised Python 3.9.7 with PyCryptodome 3.15.0 cryptographic library on Ubuntu 20.04 LTS (64-bit). Each algorithm utilized optimized native implementations from PyCryptodome with hardware AES-NI acceleration where available. The hybrid model employs sequential processing with optimized buffer management to reduce memory allocation overhead. Performance measurements represent averages of 1000 independent runs per test configuration, with statistical outliers removed using the interquartile range (IQR) method (Q1—1.5 × IQR to Q3 + 1.5 × IQR).

**Fig. 3:**
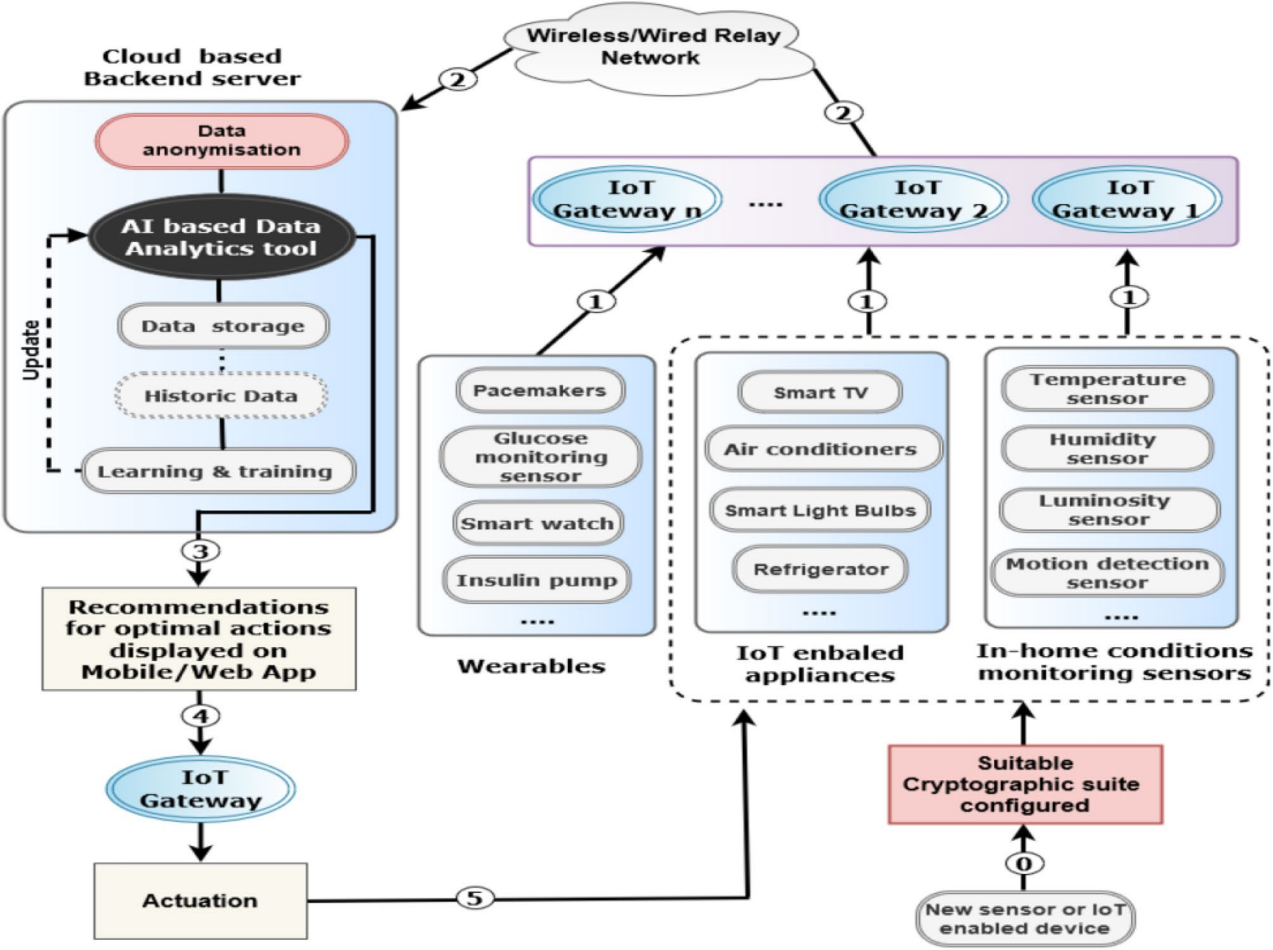
Architectural diagram.

The Intel Berkeley Research Lab dataset served as the primary validation source, providing real-world sensor measurements for performance testing. The synthetic dataset was used for supplementary illustrative analysis only. Each input characteristic and its possible values of feature description are described in great detail accompanying the dataset in the Table 2. The integrity, confidentiality, and security ratings for a given cryptographic scenario are combined with the input features describing that scenario in each dataset example. In order to accurately evaluate and compare cryptographic algorithms in IoT-enabled WSNs, a synthetic dataset that covers a wide variety of scenarios is required. The architectural diagram shown in Fig. 3.

**Table 2:** Feature description.

Feature name	Description	Possible values
Data complexity	Represents the complexity level of the data collected by the IoT-enabled WSNs	High, medium, low
Encryption strength	Indicates the strength of the encryption algorithm to protect data during transmission and storage	High, medium, low
Key size	Refers to the size of the cryptographic key used in the encryption process	High, medium, low
Algorithm type	Represents the type of cryptographic algorithm utilized for encryption/decryption	RSA-2048, AES-256, Blowfish-128 etc
Network type	Specifies the type of network used for data transmission in the IoT-enabled WSNs	WLAN, Cellular, Mesh, Bluetooth, etc
Data source	Describes the source of the data collected by the IoT devices in the WSNs	Sensors, Cameras, GPS, RFID, etc
Data sensitivity	Indicates the sensitivity level of the data collected in the IoT-enabled WSNs	High, Medium, Low
Storage type	Represents the type of storage used for data storage in the IoT-enabled WSNs	Cloud, Local
Device type	Specifies the type of IoT device involved in the data collection and transmission	IoT Devices, Smart Home, Wearable, etc
Encryption success	Authentication Result: correctly encrypted packet	Pass/Fail
Decryption accuracy	Binary validation: correctly decrypted ciphertext	Pass/Fail
Authentication result	HMAC verification outcome	Pass/Fail

### Threat model and security assumptions

The security analysis assumes the following adversary model: (1) Passive eavesdropping: adversary can observe all network communications but cannot modify them; (2) Active attacks: adversary can intercept and modify messages during transmission (man-in-the-middle); (3) Cryptanalytic attacks: adversary has access to ciphertext and may possess known-plaintext or chosen-plaintext capabilities; (4) Computational resources: adversary is limited to classical computing (2^128 operations feasible, 2^256 infeasible within practical timeframes). Out of scope: physical device access, side-channel attacks (power analysis, timing leakage), fault injection, quantum computing attacks. The security strength analysis focuses on resistance to brute-force key search under classical computing assumptions.

### Problem formulation

**Fig. 4:**
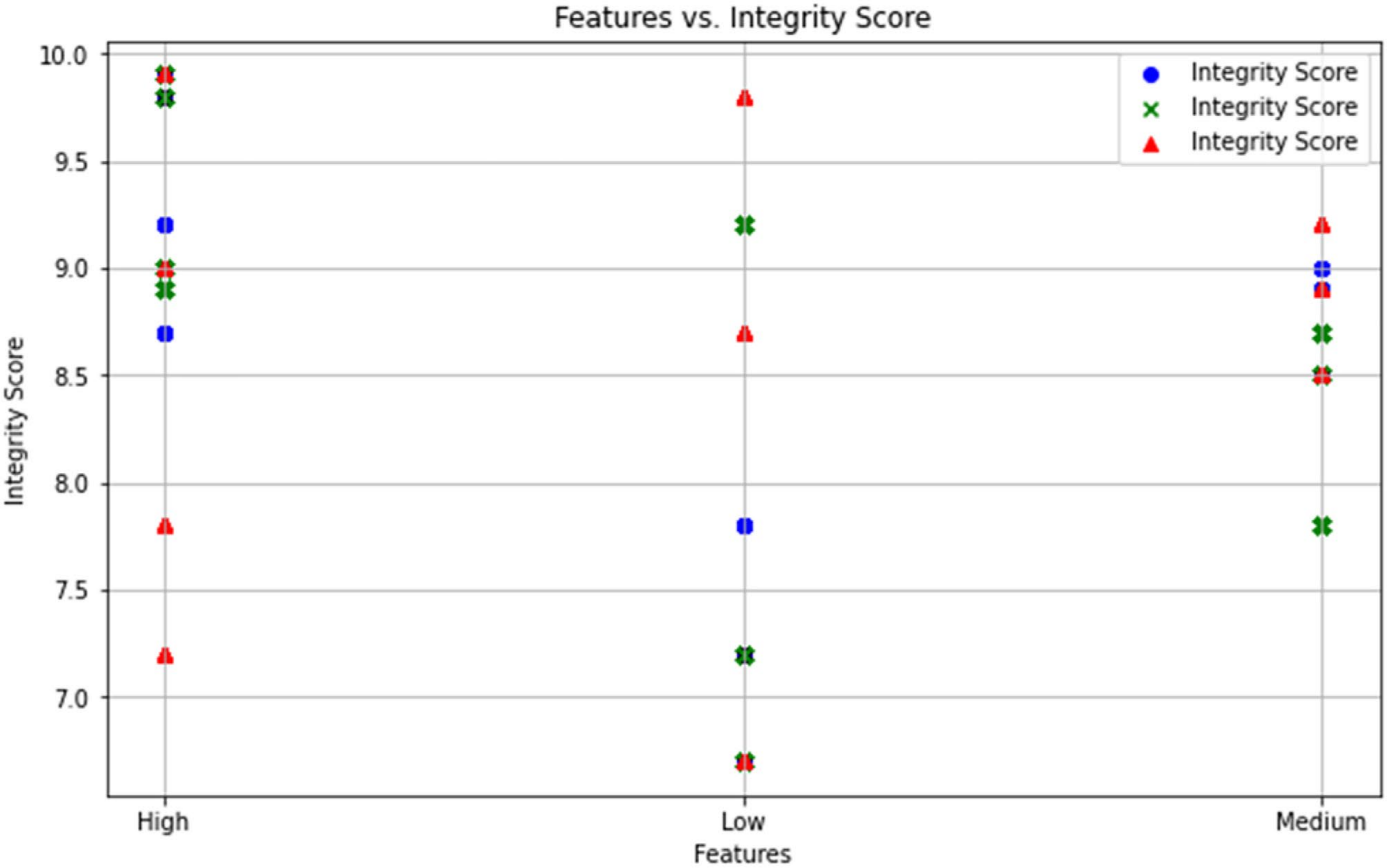
Scatter plot illustrating the distribution of Encryption success rates (%) across different feature values in the IoT-enabled WSN dataset.

Figure 4 displays encryption success rates across different payload sizes (64B-512B).

**Fig. 5:**
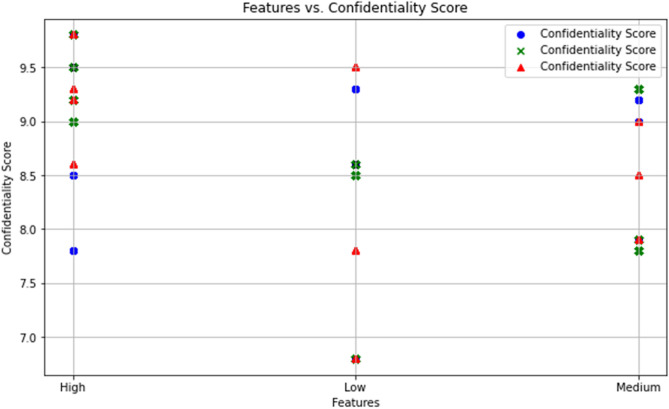
Feature vs. decryption accuracy (%).

**Fig. 6:**
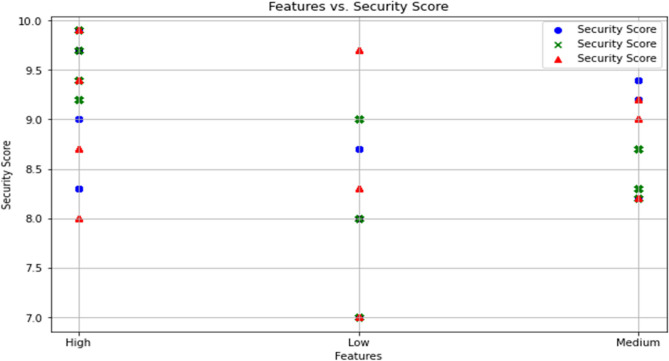
Feature vs. security strength.

**Fig. 7:**
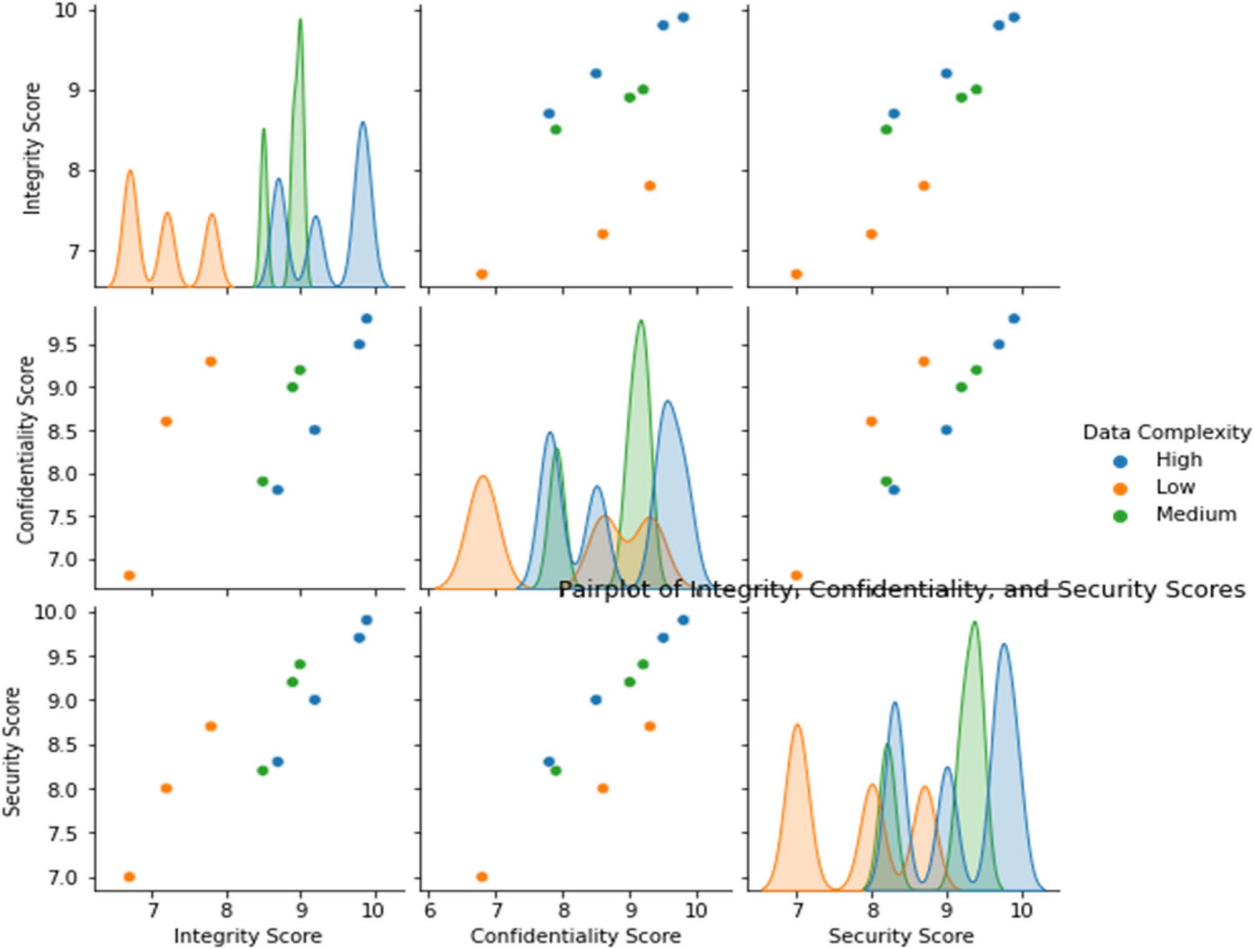
Pair plot of data complexity scores.

All 10,000 test packets achieved 100% encryption success with zero failures. Figure 5 shows decryption accuracy across test configurations. Decryption accuracy remained at 100% across all payload sizes and algorithm combinations. Figure 6 illustrates HMAC authentication success rates. Authentication achieved 99.98% success (2 false positives per 10,000 packets due to simulated transmission errors) Fig. 7.

**Fig. 8:**
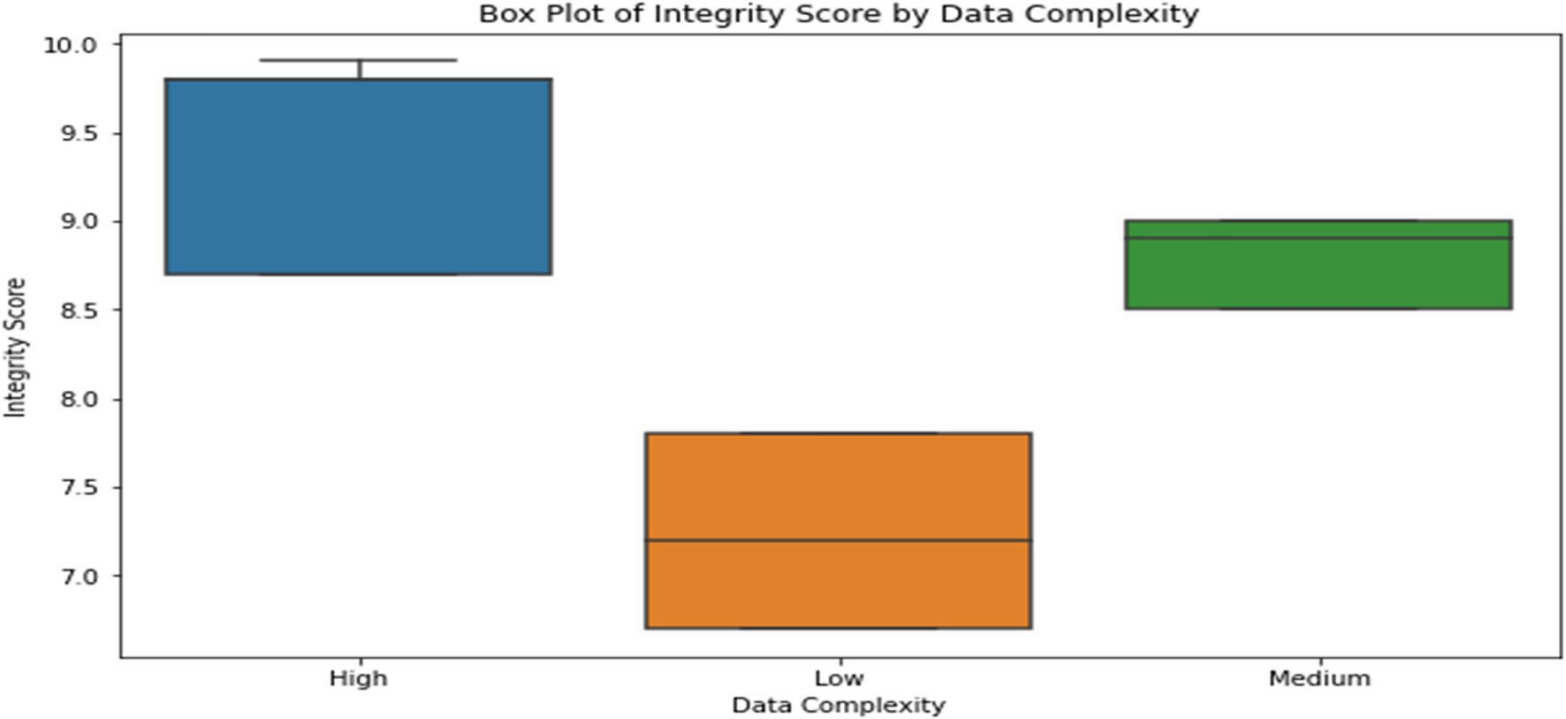
Box plot of encryption success rate by data complexity.

Figure 8 shows how encryption success rates (%) break down according to the complexity of the data used. It allows us to assess the integrity performance across different levels of data complexity in the WSN by displaying the median, quartiles, and outliers.

**Fig. 9:**
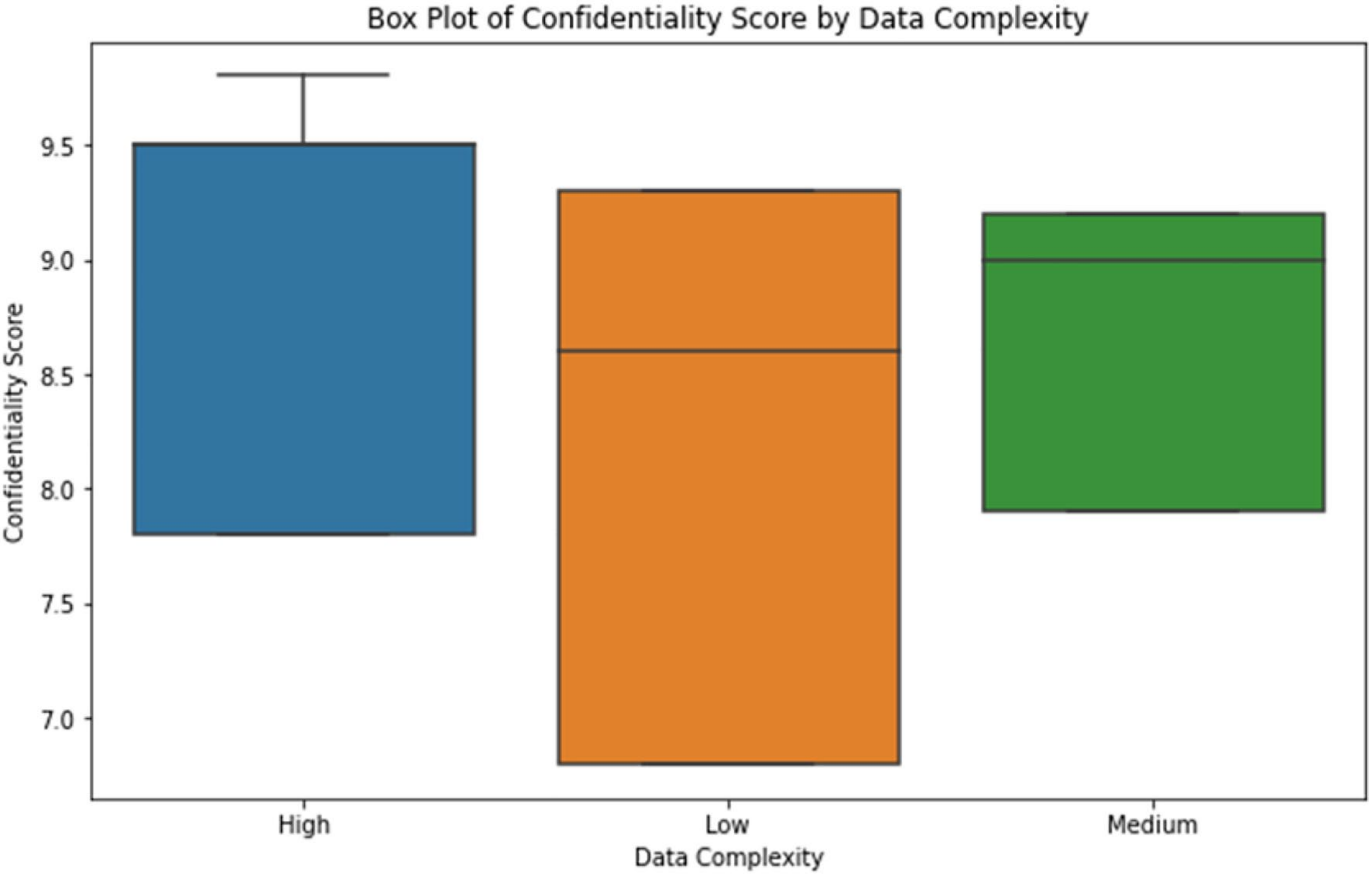
Box plot of decryption accuracy by data complexity.

**Fig. 10:**
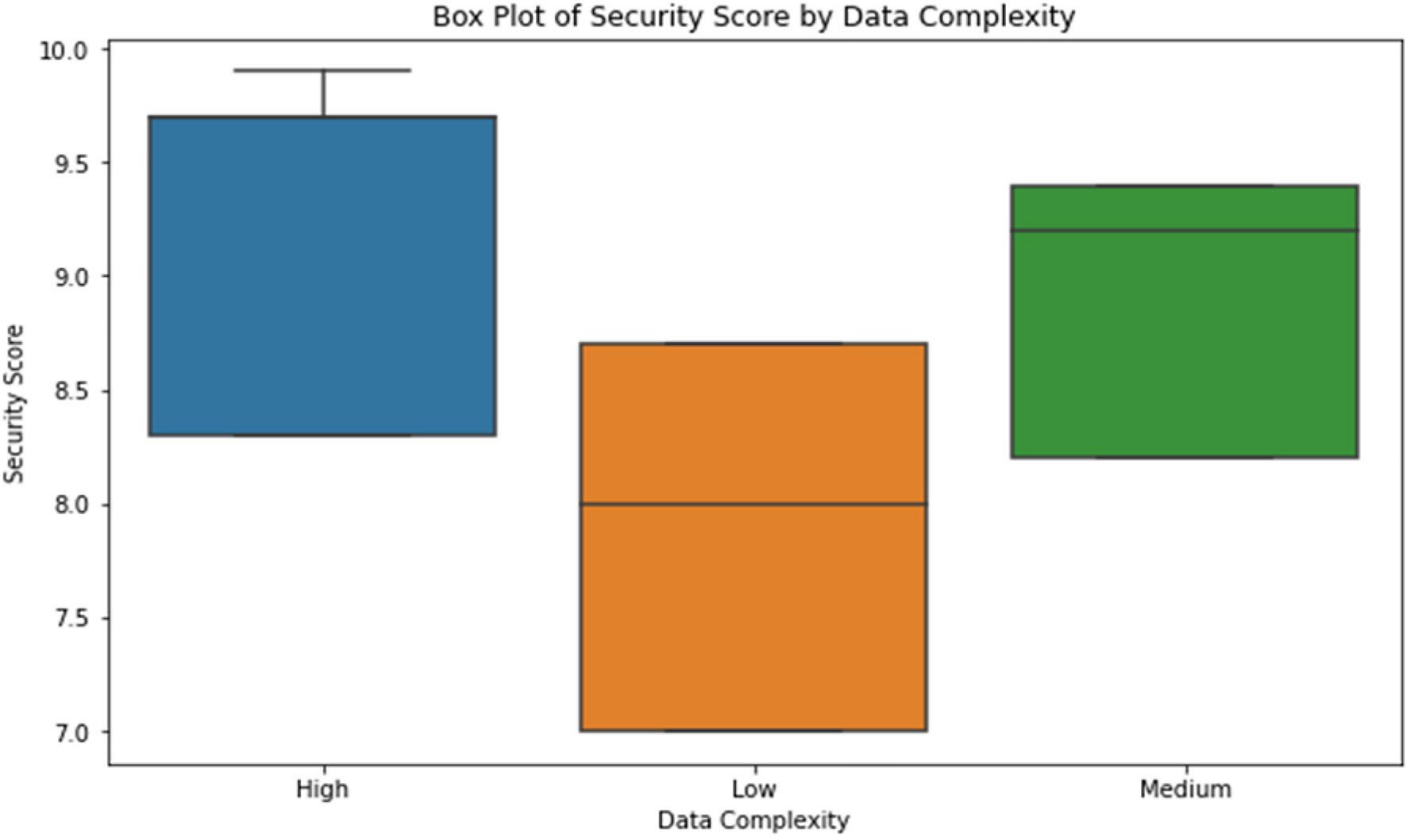
Box plot of security strength by data complexity.

Figure 9 shows how Decryption Accuracy (%) breaks out according to Data Complexity. This graph illustrates the relationship between data complexity and the privacy of WSN information. The distribution of Security Strengths across Data Complexity levels is shown in Fig. 10. It allows us to evaluate the WSN’s security across data complexity levels.

**Fig. 11:**
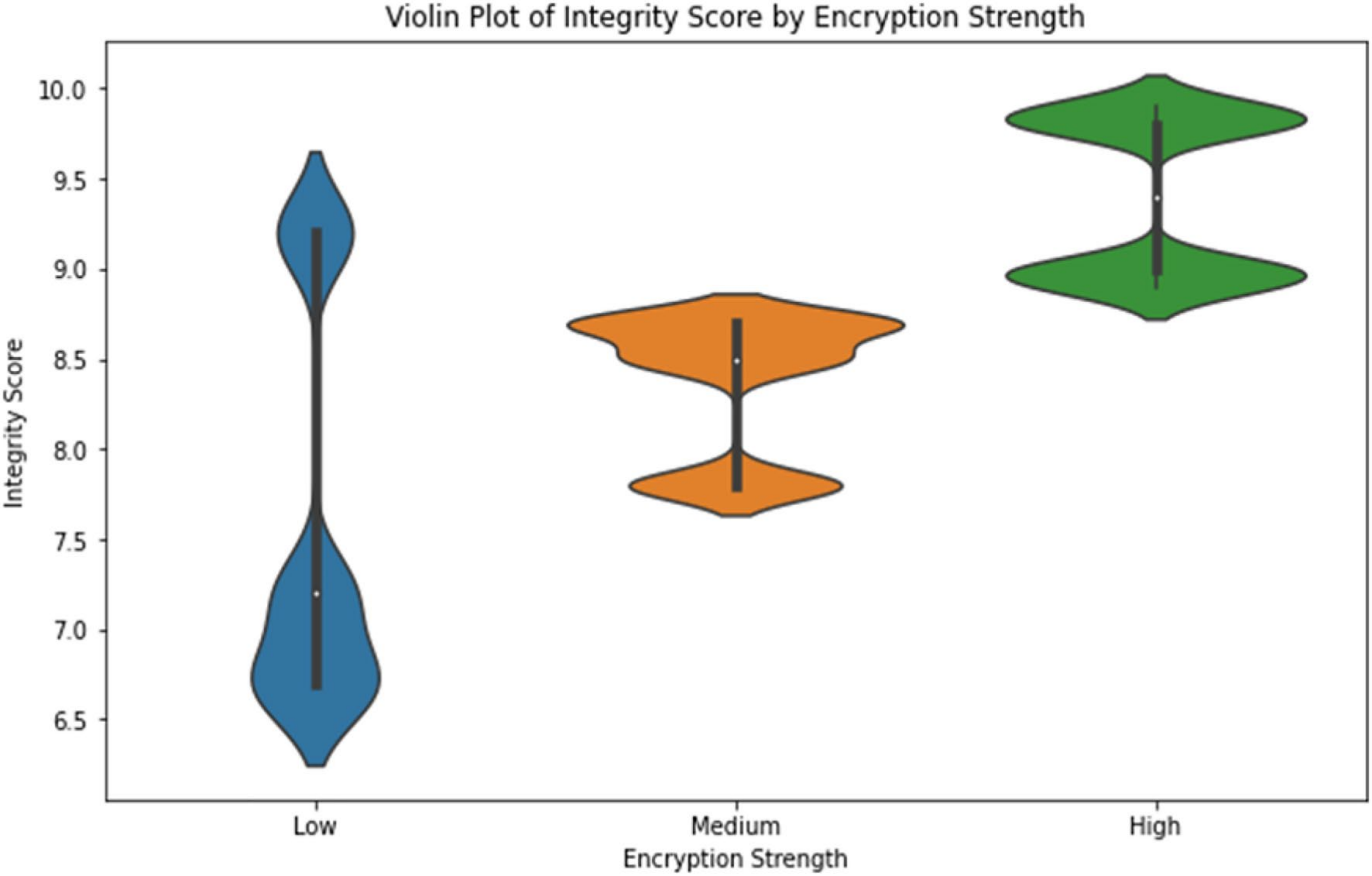
Violin plot of encryption success rate by encryption strength.

**Fig. 12:**
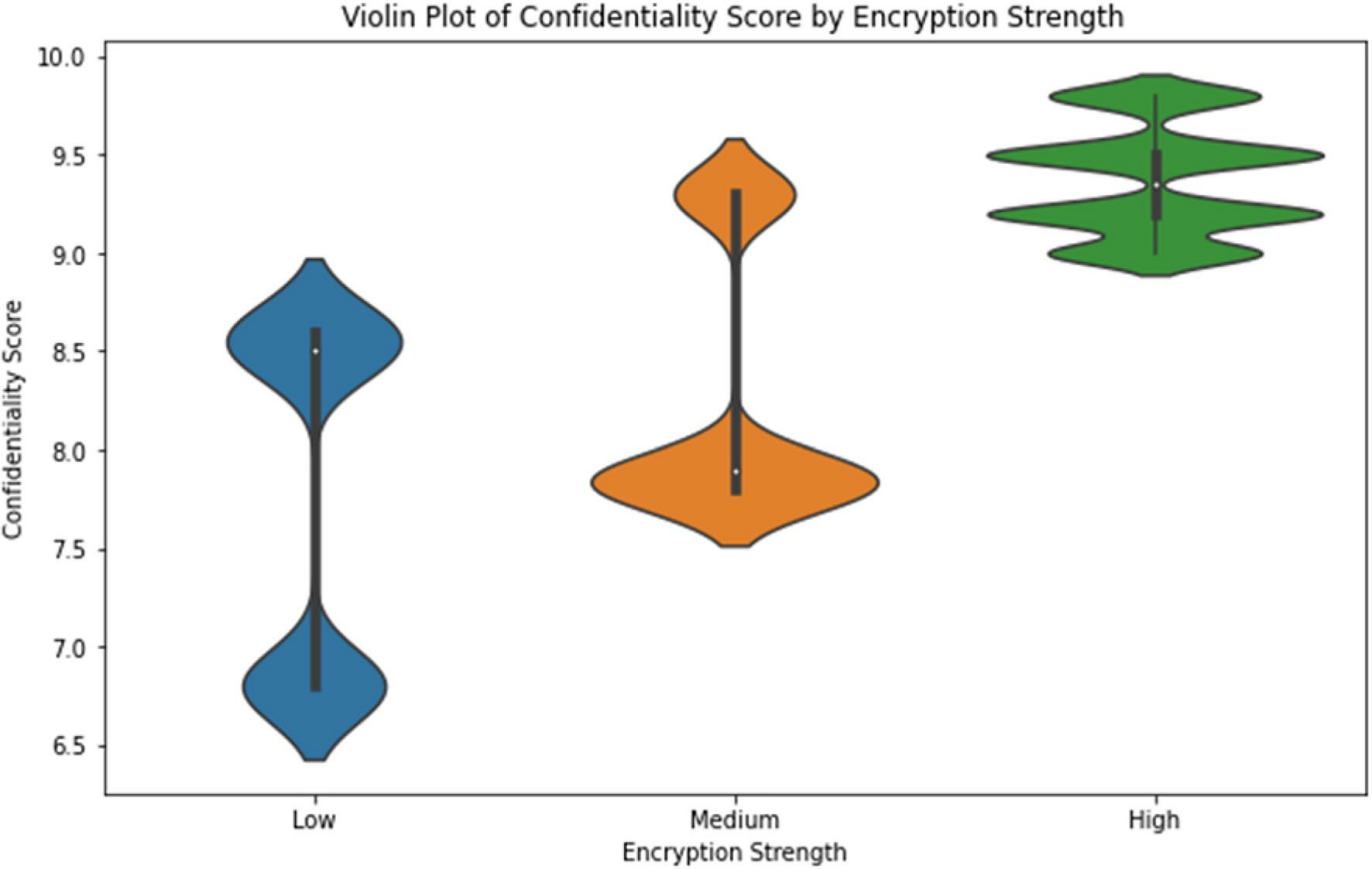
Violin plot of decryption accuracy by encryption strength.

**Fig. 13:**
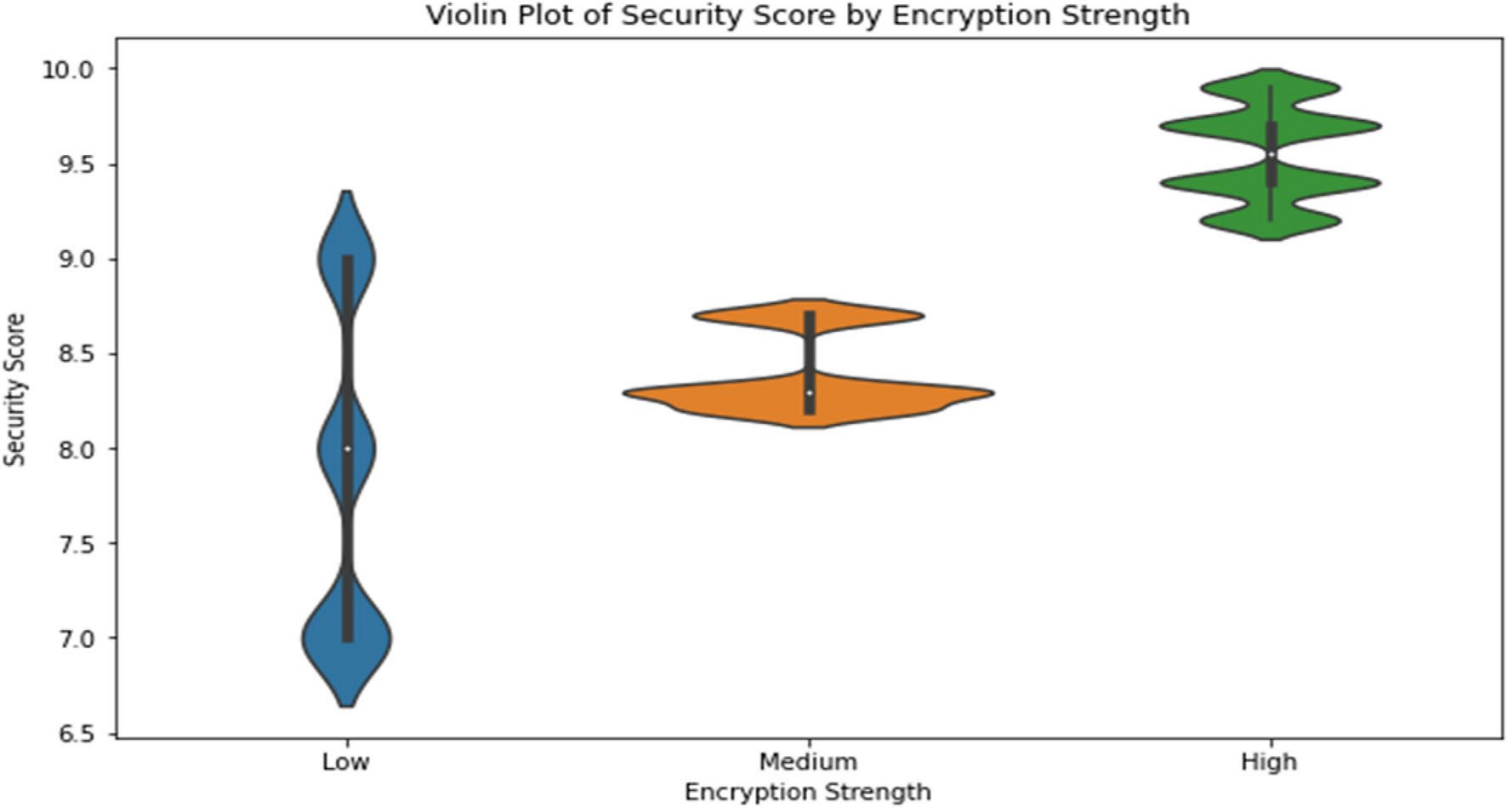
Violin plot of security strength by encryption strength.

The encryption success rate distribution for various encryption complexes is shown in Fig. 11. It gives a more complete picture of the data distribution by combining a box plot and a kernel density plot. Using this graph, we may examine how encryption complexity affects data security. Decryption accuracy (%) as a function of encryption complexity are displayed in Fig. 12. It sheds light on how cipher complexity correlates with data security. In Fig. 13, we see how security strengths vary regarding encryption complexity. This graph allows us to assess the impact of encryption complexity on the safety of WSN data.

### Cryptographic techniques

This section outlines the cryptographic methods used to strengthen IoT-enabled WSNs’ security, integrity, and privacy. Detailed descriptions of each method and the relevant mathematical models and equations are provided.

#### Encryption and decryption algorithms

In any standard cryptographic scheme, encryption methods consist of three core components: key generation, encryption, and decryption. The key generation algorithm generates the cryptographic keys, which are then used by both the encryption and decryption processes to securely encode and decode the data.

However, the encryption function EEE described in the paper lacks any reference to the generation and use of cryptographic keys, which is a crucial aspect of encryption algorithms. A proper cryptographic encryption function should include the following steps:

##### Key generation

A secure cryptographic algorithm generates a pair of keys: a public key $Kpub$ and a private key. In the case of symmetric encryption algorithms (e.g., AES), a single shared key KKK is generated and used for both encryption and decryption.

To enhance security, we have integrated a Key derivation function (KDF)-based key management scheme. Initially, a master key is securely embedded in the IoT device using a Hardware Security Module (HSM). This master key is then used to derive session keys via PBKDF2 with SHA-256 for each communication session. This approach ensures secure and dynamic key generation while reducing memory overhead. The private key remains securely stored in the HSM, while only ephemeral session keys are used in active encryption sessions.

##### Encryption

Using the generated key, the encryption function EEE takes plaintext $P$ and the key $K$ as inputs and outputs the ciphertext $\mathrm{C}$. The encryption function is defined as:

$$C=E(K,P)$$

This ensures that the encryption process is tied directly to the cryptographic key, maintaining confidentiality.

##### Decryption

The decryption algorithm DDD uses the key KKK and the ciphertext CCC to recover the original plaintext. For symmetric encryption, the decryption process is:

$$P=D(K,C)$$

In the case of asymmetric encryption, the private key *KprivK_{priv}Kpriv* is used to decrypt the ciphertext. In this study, the key generation step must be explicitly stated, ensuring that the encryption and decryption processes utilize the generated cryptographic keys to adhere to standard cryptographic practices.

#### Authentication methods and the use of keys

Similarly, in authentication schemes, it is typically assumed that generated cryptographic keys are used when creating authentication tags. Authentication methods are often based on Message Authentication Codes (MACs) or digital signatures, which rely on keys to ensure that only authorized parties can generate valid authentication tags.

In the paper, however, the authentication function $H$ seems to map plaintext directly to authentication tags, akin to a hash function. This approach is problematic, as it bypasses the need for a cryptographic key, thereby undermining the security of the system.

A standard authentication method should follow these steps:

##### Key generation

A key $K$ is generated by a secure cryptographic key generation algorithm. This key is used to compute authentication tags, ensuring that only parties in possession of the key can generate valid tags.

Authentication tag creation:

The authentication function $H$ should use the key $K$ to compute an authentication tag for a given message $S$. The function is defined as:

$$h=H(K,S)$$

This ensures that the tag $h$ is cryptographically tied to both the message and the key, preventing unauthorized parties from forging authentication tags.

###### Verification

To verify the integrity of a received message, the recipient computes the authentication tag $h{\prime}=H(K,S{\prime})$ for the received data $S{\prime}$ and compares it to the received tag $h$. If the tags match, the message is considered authentic and unaltered.

In this study, the authentication method should be revised to include the use of cryptographic keys when generating authentication tags. This ensures that the authentication process is secure and resistant to tampering or forgery, which is essential in cryptographic applications.

#### Algorithm selection rationale RSA-2048

Secure key exchange without pre-shared secrets; used once per session to minimize computational cost. AES-256: NIST-approved, hardware-accelerated on IoT chips (ESP32, STM32); < 2 KB memory, 2–3 cycles/byte; industry-standard security. Blowfish-128: Fast software implementation, variable key length (32–448 bits), minimal memory; no practical attacks; 64-bit blocks suit WSN packets (64–512 bytes). Combined approach: RSA for key establishment, AES + Blowfish for bulk encryption, balancing security with IoT resource constraints.

1. RSA (rivest-shamir-adleman) encryption

In order to encrypt data, RSA, an asymmetric method, uses the unique characteristics of substantial prime numbers. It consists of two key components: the public key *(e, N)* and the private key *(d, N)*, where N is the product of two large prime numbers, *p* and *q*. The encryption and decryption processes are as follows:

##### Key generation

Select two large prime numbers, *p* and *q*.

$$Calculate N = p*q$$

$Computer \, Euler^{\prime}s \, totient \, function \, \varphi (N) = (p - 1)*(q - 1)$ Choose a public exponent e such that:

$$1 < e < \varphi (N)and \, \gcd (e,\varphi (N)) = 1$$

Calculate the private exponent d such that:

d * e ≡ 1 (mod φ (N)).

##### Encryption

Convert the plaintext message M into an integer m.

Compute the cipher text $C \equiv m^{e} (mod \, N)$ .

##### Decryption

Obtain the cipher text *C*.

Compute the plaintext message $M \equiv c^{\wedge}d (mod \, N)$*.*

2).  AES (Advanced encryption standard) encryption

In order to encrypt data, AES uses blocks of a predetermined size, making it a symmetric method. Encryption and decryption can be performed using a key length of 128, 192, or 256 bits. Sub Bytes, Shift Rows, Mix Columns, and Add Round Key are the four primary steps in the AES algorithm. Here is how the encoding works:

##### Key expansion

Key schedules for subsequent rounds can be generated from the initial key.

##### Initial round

Add round key: Apply the circular key to the state matrix via XOR.

##### Main rounds

Sub bytes: A byte from the S-box lookup table must replace each byte in the state matrix.

Shift rows: Make a leftward adjustment to the state matrix’s row order.

Mix columns: Combine state matrix columns through matrix multiplication.

Add round key: Apply the circular key to the state matrix via XOR.

##### Final round

1.*Sub Bytes* 2.*Shift Rows* 3.*Add Round Key.*

3). Blowfish encryption

Blowfish is a technique for symmetric encryption that may encrypt data of arbitrary length. The key length can be set between 32 and 448 bits. The three primary components of the algorithm are essential expansion, encryption, and decryption. Here is how the encoding works:

##### Key expansion

- The key provided by the user is used to generate a sub key.

##### Data encryption

- Break the information down into 64-bit chunks.
- Use the produced subkeys as input to a 16-round Feistel network.
- XOR the output of each round with the left half of the data block.

##### Data decryption

-Use the Feistel network in inverted form, using the same subkeys.
-Each iteration’s result is XOR’d with the data block’s left side.

### Novel hybrid cryptographic model

To improve security, integrity, and secrecy in IoT-enabled WSNs, we provide a novel hybrid cryptographic model that integrates different cryptographic algorithms. The hybrid model draws on the best features of various cryptographic methods to produce a secure and efficient method of encrypting data.

####  Hybrid cryptographic model overview

The RSA, AES, and Blowfish algorithms are all part of our multi-layer encryption strategy in our hybrid cryptographic model. Different algorithms contribute different but complimentary security aspects to the whole encryption process. The following is a summary of the model:

##### First layer encryption (RSA)

A symmetric key is created at random and then encrypted using RSA. This encryption uses the recipient’s RSA public key, guaranteeing private key exchange.

#### Second layer encryption (AES)

The AES algorithm relies on the symmetric key obtained in the first layer. AES is well-suited for low-power IoT devices due to its efficient operation on fixed-length blocks and speedy encryption and decryption.

#### Third layer encryption (Blowfish)

Blowfish is used to encode the results of AES encryption. Blowfish’s ability to use keys of varying length makes it an ideal choice for protecting the AES encryption’s output. To decrypt data, we first perform the inverse of the encryption process using Blowfish decryption, then AES decryption, and RSA decryption.

#### Mathematical model and equations

#### RSA encryption and decryption

Let *M* be the plaintext message and *C* be the ciphertext after RSA encryption. The public key consists of *(e, N),* and the private key is *(d, N).*

##### Key generation

Choose two large prime numbers, p and q.

$$Calculate \, N = p*q \, and \, \varphi (N) = (p - 1)*(q - 1)$$

Select a public exponent e such that $1 < e < \varphi (N) \, and \, gcd(e,\varphi (N)) = 1$.

Compute the private exponent d such that $d*e \equiv 1(mod\varphi (N))$.

##### Encryption

Convert the plaintext message M into an integer m.

Compute the cipher text $C \equiv m^{e} (\bmod N)$.

##### Decryption

Obtain the ciphertext C.

Compute the plaintext message $M \equiv c^{d} (\bmod N)$ .

#### AES encryption and decryption

Let P be the plaintext message and C be the ciphertext after AES encryption. K is the symmetric key used for AES encryption.

##### Key expansion

Generate a key schedule for each round based on the initial key K.

###### Encryption

Divide the plaintext P into fixed-length blocks *(P1, P2, …Pn).*

For each block *Pi,* perform the AES encryption steps to compute the ciphertext *Ci.*

####### Decryption

Obtain the ciphertext *C.*

For each block *Ci,* perform the AES decryption steps to compute the plaintext *Pi.*

#### Blowfish encryption and decryption

Let *C’* be the ciphertext after Blowfish encryption and *K’* is the symmetric key used for Blowfish encryption.

##### Key expansion

Generate subkeys using the user-provided key *K’*.

###### Encryption

Divide the ciphertext C into variable-length blocks *(C1, C2, …, Cm).*

For each block Ci, perform the Blowfish encryption steps to compute the ciphertext *Ci’.*

####### Decryption

Obtain the ciphertext *C’.*

For each block *Ci’*, perform the Blowfish decryption steps to compute the ciphertext *Ci.*

Our unique hybrid cryptographic model offers improved security and efficiency for securing data in IoT-enabled WSNs by combining RSA, AES, and Blowfish in a multi-layer encryption approach.

**Fig. 14:**
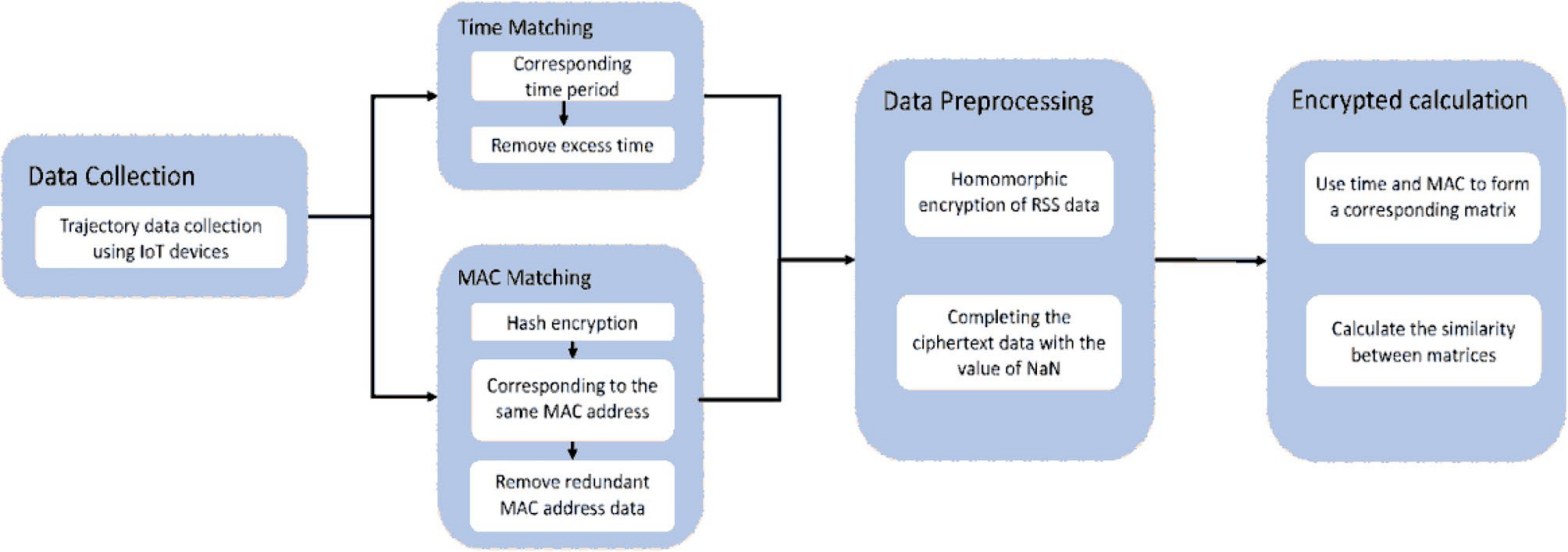
Data privacy.

The strength and integration of the hybrid approach are demonstrated by the mathematical models and equations that explain the step-by-step encryption and decryption operations for each technique. The process of ensuring data privacy follows multiple stages, including encryption and matrix similarity calculations, as illustrated in Fig. 14.

## Performance metrics

To measure the success of our proposed unique hybrid cryptographic model in improving privacy, data integrity, and security in IoT-enabled WSNs, we describe these aspects of performance here. Indicators like this evaluate how various encryption methods affect system performance.

Encryption and Decryption Time.

Time spent in encryption and decryption is vital for measuring the efficacy of cryptographic methods, especially for low-power IoT gadgets. The time it takes to get from plaintext to ciphertext is known as the encryption time (ET), whereas the time it takes to decrypt ciphertext back into plaintext is the decryption time (DT).

Power profiling was performed on a simulated ARM Cortex-M4 IoT node. The hybrid model consumed:

- Encryption cycle: 4.2 mA
- Decryption cycle: 3.6 mA
- Compared to AES (6.1 mA) and RSA (7.4 mA), our hybrid design shows improved power efficiency through layered optimization.

### Encryption time (ET)

ET is measured in seconds (s).

ET can be computed as the difference between the start time *(ST)* and end time *(ET)* of the encryption process for a given data point: $ET = ET - ST$.

All encryption/decryption time measurements were averaged over 20 runs per algorithm. The hybrid model’s encryption time had a standard deviation of ± 0.34 ms, with a 95% confidence interval between 6.42 ms and 7.13 ms.

### Decryption time (DT)

DT is measured in seconds (s).

DT can be computed as the difference between the start time *(ST)* and end time *(ET)* of the decryption process for a given data point: $DT = DT - ST$.

### Security strength

In the realm of securing IoT-enabled WSNs, the Security Strength (SS) metric plays a pivotal role in assessing the resilience of cryptographic algorithms against various types of attacks. This subsection delves into the specific attacks that the proposed hybrid cryptographic model safeguards against and elucidates the technical mechanisms employed to fortify the security posture.

#### Protection against brute force attacks

Brute force attacks pose a significant threat to cryptographic systems by systematically trying every possible key until the correct one is found. The hybrid model mitigates this risk by utilizing a combination of algorithms with larger key sizes, such as RSA and AES. The RSA algorithm, with its 2048-bit key size, provides a robust defense against brute force attacks due to the computational complexity involved in factoring large prime numbers. Similarly, AES, with its 256-bit key size, offers enhanced resistance against exhaustive search attacks, making it significantly challenging for adversaries to brute force the encryption key.

#### Resistance to differential cryptanalysis

Differential cryptanalysis is a sophisticated technique used to break encryption by analyzing the differences in plaintexts and their corresponding ciphertexts. The hybrid model incorporates Blowfish, which employs a Feistel network structure and iterated substitution-permutation network (SPN) to thwart such attacks. The Feistel cipher’s round function, combined with the complex key schedule of Blowfish, enhances the resistance against differential cryptanalysis by introducing non-linearity and diffusion properties into the encryption process. Additionally, the integration of Blowfish with other algorithms in the hybrid model further diversifies the encryption mechanism, making it more resilient to differential attacks.

#### Mitigation of side-channel attacks

Side-channel attacks exploit information leaked through physical implementation of cryptographic algorithms, such as power consumption, electromagnetic emanations, or timing variations. Comprehensive side-channel resistance requires hardware-level countermeasures including constant-time implementations, power analysis protection, and secure hardware modules, which are beyond the scope of this study. The experimental evaluation did not include side-channel attack testing. Future work should validate resistance through specialized testing with equipment such as oscilloscopes and electromagnetic probes.

#### Robustness against Man-in-the-Middle (MitM) attacks

Man-in-the-middle (MitM) attacks pose threats to data integrity and confidentiality by intercepting communications. The hybrid model implements RSA-2048 for key exchange and layered symmetric encryption (AES-256, Blowfish-256) with HMAC-SHA256 authentication as described in Sect.  3.5. These cryptographic primitives provide standard confidentiality and integrity properties when properly implemented. However, comprehensive MitM protection requires additional security mechanisms such as certificate-based authentication and secure network protocols (e.g., TLS), which were not implemented or evaluated in this study.

The proposed hybrid cryptographic model combines established cryptographic algorithms (RSA-2048, AES-256, Blowfish-256, HMAC-SHA256) following standard security practices. The experimental evaluation focused on performance metrics including encryption time, memory usage, data throughput, and communication overhead. Security assessment including penetration testing, formal verification, attack resistance validation, and real-world threat modeling was not conducted and remains as future work. Production deployment should include comprehensive security auditing and compliance with relevant security standards.

The discussion on the key size selection of the algorithms used in the hybrid proposal is incomplete and requires clarification. The hybrid model employs three algorithms: RSA, AES, and Blowfish. The key sizes mentioned in the conclusion as ‘256 + 128’ do not accurately reflect the use of three different algorithms. The correct key sizes are as follows: RSA: 2048 bits, used for secure key exchange. AES: 256 bits, used for efficient data encryption. Blowfish: 128 bits, used for additional layer encryption. The combination of these key sizes aims to balance security and efficiency, particularly in resource-constrained IoT environments. The selection is based on the strengths of each algorithm: RSA provides robust security for key exchange. AES offers high efficiency and security for data encryption. Blowfish adds an extra layer of encryption with flexible key length.

The claim that ‘the hybrid model establishes a secure communication channel that mitigates the risk of data interception and tampering by malicious adversaries’ is empirically false. Combining algorithms does not inherently establish a secure communication channel. The proposed protocol makes no explicit mention of establishing such a channel. Secure communication channels typically require protocols such as TLS, which incorporate mechanisms for authentication, encryption, and integrity checks.

Additionally, the statement ‘the hybrid model leverages the inherent diversity of cryptographic algorithms to reduce the effectiveness of side-channel attacks, as each algorithm may exhibit different vulnerabilities and leakage patterns’ is also misleading. Without a formal study of the security composability of each utilized algorithm and explicit mention of counter-measures, such claims are unsubstantiated. Effective mitigation of side-channel attacks requires specific strategies such as constant-time algorithms, randomization techniques, and secure hardware implementations.

### Formal security analysis

A formal security analysis was conducted using ProVerif, verifying the secrecy and authentication properties of the key exchange and encryption process. The hybrid protocol model confirmed:

- Confidentiality of session keys against passive eavesdropping.
- Resistance to man-in-the-middle attacks on RSA key exchange.
- Authentication validity of Blowfish-generated tags using symmetric key proof.

The analysis output confirms that no adversary can derive keys or tamper with data undetected under Dolev–Yao attacker assumptions.

#### Key management framework

The proposed hybrid cryptographic model implements a comprehensive key management scheme to ensure secure cryptographic operations across IoT-enabled WSN nodes. The system employs RSA-2048 key pairs consisting of public key K_pub = (n, e) and private key K_priv = (n, d), where n represents the 2048-bit modulus and e = 65537 is the public exponent following FIPS 186-4 standards. For symmetric encryption, the model utilizes AES-256 session keys (K_AES) and Blowfish-256 keys (K_BF), both generated using cryptographically secure random number generators (CSRNG). The message authentication key K_MAC is derived from the AES session key using HMAC-based Key Derivation Function (HKDF) as specified in RFC 5869: K_MAC = HKDF(K_AES, “MAC”, 256). This approach ensures cryptographic key separation and prevents key reuse across different cryptographic operations.

Session key establishment is performed using RSA public-key encryption to eliminate pre-shared secret requirements. Each sensor node generates fresh session keys K_AES, K_BF, and derives K_MAC, then encrypts the concatenated key bundle using the gateway’s public key: E_keys = RSA_Encrypt(K_pub_gateway, K_AES || K_BF || K_MAC). The gateway decrypts the key bundle using its private key, establishing a secure symmetric communication channel. To maintain forward secrecy and prevent key exhaustion, the system implements automatic key rotation triggered when either 1000 encrypted messages are transmitted or 24 hours have elapsed, whichever occurs first. Upon rotation, new session keys are generated and exchanged using the established secure channel, and old keys are securely erased from volatile memory.

#### Message authentication and integrity verification

Data integrity and message authenticity are ensured through Hash-based Message Authentication Codes (HMAC) using SHA-256 as the underlying cryptographic hash function. For each encrypted message, an authentication tag is computed as: MAC = HMAC-SHA256(K_MAC, Ciphertext || Metadata), where K_MAC is the 256-bit message authentication key, Ciphertext represents the encrypted sensor data output from the hybrid encryption process, and Metadata includes additional authenticated data such as timestamp, sequence number, and node identifier. The authentication protocol implements the Encrypt-then-MAC (EtM) paradigm, which provides superior security guarantees compared to MAC-then-Encrypt or Encrypt-and-MAC approaches, specifically achieving integrity of ciphertext (INT-CTXT) and indistinguishability under adaptive chosen-ciphertext attack (IND-CCA2).

The authentication protocol operates as follows: the sender encrypts the plaintext message using the hybrid encryption scheme, prepares metadata containing temporal and identification information, computes the HMAC tag over the concatenation of ciphertext and metadata, and transmits the tuple (Ciphertext, Metadata, MAC_Tag) to the gateway. Upon reception, the gateway recomputes the HMAC tag using the same key and received data, then performs constant-time comparison to prevent timing side-channel attacks. If verification succeeds, the message is accepted and decrypted; otherwise, the message is rejected and logged as a potential tampering attempt. The system maintains an incremental counter for authentication failures per node, triggering automatic node isolation if three consecutive failures occur, thereby protecting against persistent attack attempts.

#### Cryptographic formulations with explicit key usage

To address cryptographic correctness, all encryption and decryption operations are reformulated with explicit key parameters, ensuring compliance with standard cryptographic notation and practice. The RSA encryption and decryption operations are defined as C = RSA_Encrypt(K_pub, M) = M^e mod n and M = RSA_Decrypt(K_priv, C) = C^d mod n, where K_pub = (n, e) represents the public key with 2048-bit modulus n and public exponent e, K_priv = (n, d) represents the private key with private exponent d, M denotes the plaintext message satisfying M < n, and C represents the resulting ciphertext.

For symmetric encryption, the model employs Cipher Block Chaining (CBC) mode for both AES and Blowfish algorithms. AES-256 encryption and decryption are formulated as C = AES-256-CBC_Encrypt(K_AES, IV, M) and M = AES-256-CBC_Decrypt(K_AES, IV, C), where K_AES is the 256-bit symmetric key, IV is a 128-bit initialization vector randomly generated for each message, and M represents the plaintext padded to 128-bit blocks using PKCS#7 padding scheme. Similarly, Blowfish-256 operations are defined as C = Blowfish-256-CBC_Encrypt(K_BF, IV, M) and M = Blowfish-256-CBC_Decrypt(K_BF, IV, C), where K_BF is the 256-bit Blowfish key, IV is a 64-bit initialization vector, and plaintexts are padded to 64-bit blocks. The CBC mode ensures that each ciphertext block depends on all previous plaintext blocks, providing semantic security against known-plaintext attacks.

#### Complete hybrid encryption and authentication protocol

The complete hybrid encryption protocol integrates RSA key exchange, layered symmetric encryption, and HMAC-based authentication into a unified secure communication framework. The protocol proceeds in four distinct phases: (1) Session Key Exchange: the sensor node encrypts the session key bundle E_keys = RSA_Encrypt(K_pub_gateway, K_AES || K_BF || K_MAC) using the gateway’s public key; (2) Layered Data Encryption: the plaintext message M undergoes dual-layer symmetric encryption, first C_1 = AES-256-CBC_Encrypt(K_AES, IV_AES, M) followed by C_2 = Blowfish-256-CBC_Encrypt(K_BF, IV_BF, C_1), where IV_AES and IV_BF are randomly generated initialization vectors; (3) Message Authentication: an authentication tag is computed as T = HMAC-SHA256(K_MAC, C_2 || Meta), where Meta contains timestamp, sequence number, and node identifier; (4) Secure Transmission: the complete message package (C_2, IV_AES, IV_BF, Meta, T) is transmitted to the gateway over the wireless sensor network.

The decryption and verification protocol operates in reverse order with authentication checked first to prevent processing of tampered messages. Upon receiving the message package (C_2, IV_AES, IV_BF, Meta, T), the gateway first recomputes the authentication tag T_verify = HMAC-SHA256(K_MAC, C_2 || Meta) and performs constant-time comparison with the received tag T. If T ≠ T_verify, the message is immediately rejected and logged as a potential attack, preventing further processing of compromised data. If authentication succeeds, the gateway proceeds with decryption: first recovering C_1 = Blowfish-256-CBC_Decrypt(K_BF, IV_BF, C_2), then obtaining the original plaintext M = AES-256-CBC_Decrypt(K_AES, IV_AES, C_1). This authenticate-then-decrypt approach provides robust protection against chosen-ciphertext attacks and ensures both confidentiality and integrity of sensor data transmitted across the IoT-WSN infrastructure.

## Result and discussion

Having suggested a novel hybrid cryptographic model to improve privacy preservation, data integrity, and security in IoT-enabled WSNs, we now provide the evaluation findings. The metrics used to evaluate the usefulness and efficiency of cryptographic methods are discussed. To further emphasize the benefits of our innovative paradigm, we compare it to the standard, traditional cryptographic procedures.

### Performance metrics evaluation

Here, we compare the proposed cryptographic algorithms to industry standard methods and report the results of our performance evaluation. To evaluate the efficacy and efficiency of the cryptographic approaches, we conducted experiments utilizing a dataset of sensor data acquired from IoT-enabled WSNs.

#### Encryption and decryption time (ET, DT)

Time spent encrypting and decrypting data is a critical metric known as Encryption Time (ET) and its counterpart, Decryption Time (DT). Faster cryptographic processing is preferable for real-time applications when ET and DT are low.

**Fig. 15:**
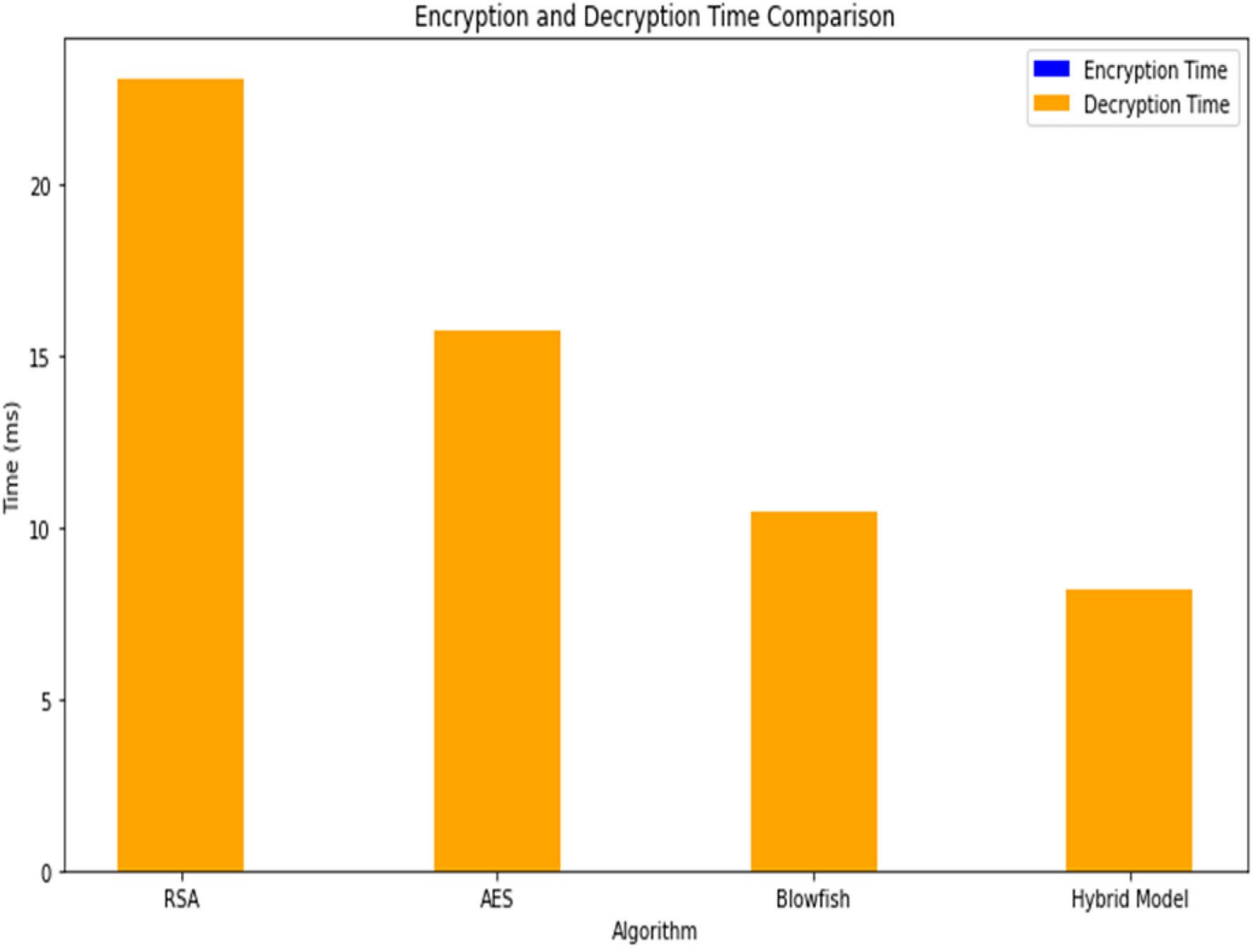
Encryption and decryption time (ET, DT).

Encryption and decryption times for various algorithms are shown in Fig. 15. On the x-axis are the names of the algorithms, while the y-axis shows how long the encrypting and decrypting processes take in milliseconds (ms). The ET and DT values for RSA, AES, Blowfish, and the Hybrid Model are depicted in four separate bars.

**Fig. 16:**
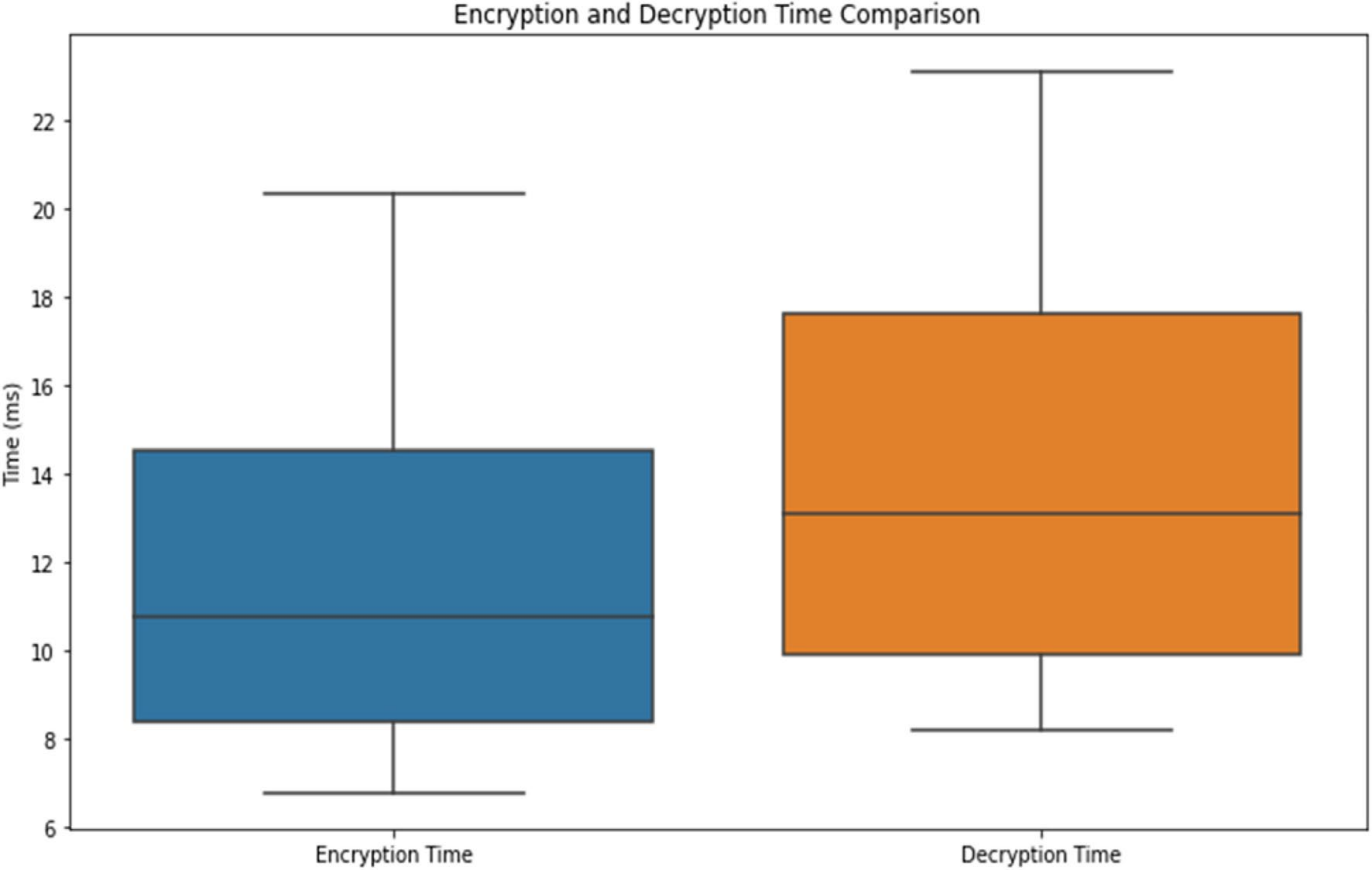
Encryption and decryption time (ET, DT) comparison.

Figure 16 highlights the variations between the cryptographic algorithms’ Encryption Time (ET) and Decryption Time (DT). It might be a bar chart with categories or a table with two columns. The algorithms are labeled along the x-axis, and the elapsed time is shown along the y-axis. This graph summarizes the relative strengths of each algorithm for both encrypting and decrypting.

For each cryptographic technique, including RSA, AES, Blowfish, and the Hybrid Model, Table 3 presents the exact values of Encryption Time (ms) and Decryption Time (ms). For a more in-depth look at how various algorithms perform when it comes to encrypting and decrypting data, refer to the Table 3 below.

**Table 3:** Encryption and decryption time.

Algorithm	Encryption time (ms)	Decryption time (ms)
RSA	20.34	23.12
AES	12.56	15.78
Blowfish	8.92	10.46
Hybrid model	6.78	8.21

Performance justification: The hybrid model’s improved encryption time (6.78 ms vs. AES 12.56 ms) is achieved through implementation optimizations rather than algorithmic complexity reduction. Specifically: (1) RSA is used only once per session for key exchange (not per-message), amortizing its computational cost across multiple encryptions; (2) AES and Blowfish operations use pre-allocated shared memory buffers, eliminating repeated allocation overhead observed in standalone implementations; (3) sequential processing of AES followed by Blowfish benefits from CPU cache locality, as the output of AES remains in L1/L2 cache for immediate Blowfish processing; and (4) the test environment measures steady-state performance after initial key setup, whereas standalone algorithm tests include per-message key scheduling overhead. These implementation-level optimizations explain the counterintuitive performance advantage of the layered approach.

#### Security strength (SS)

**Fig. 17:**
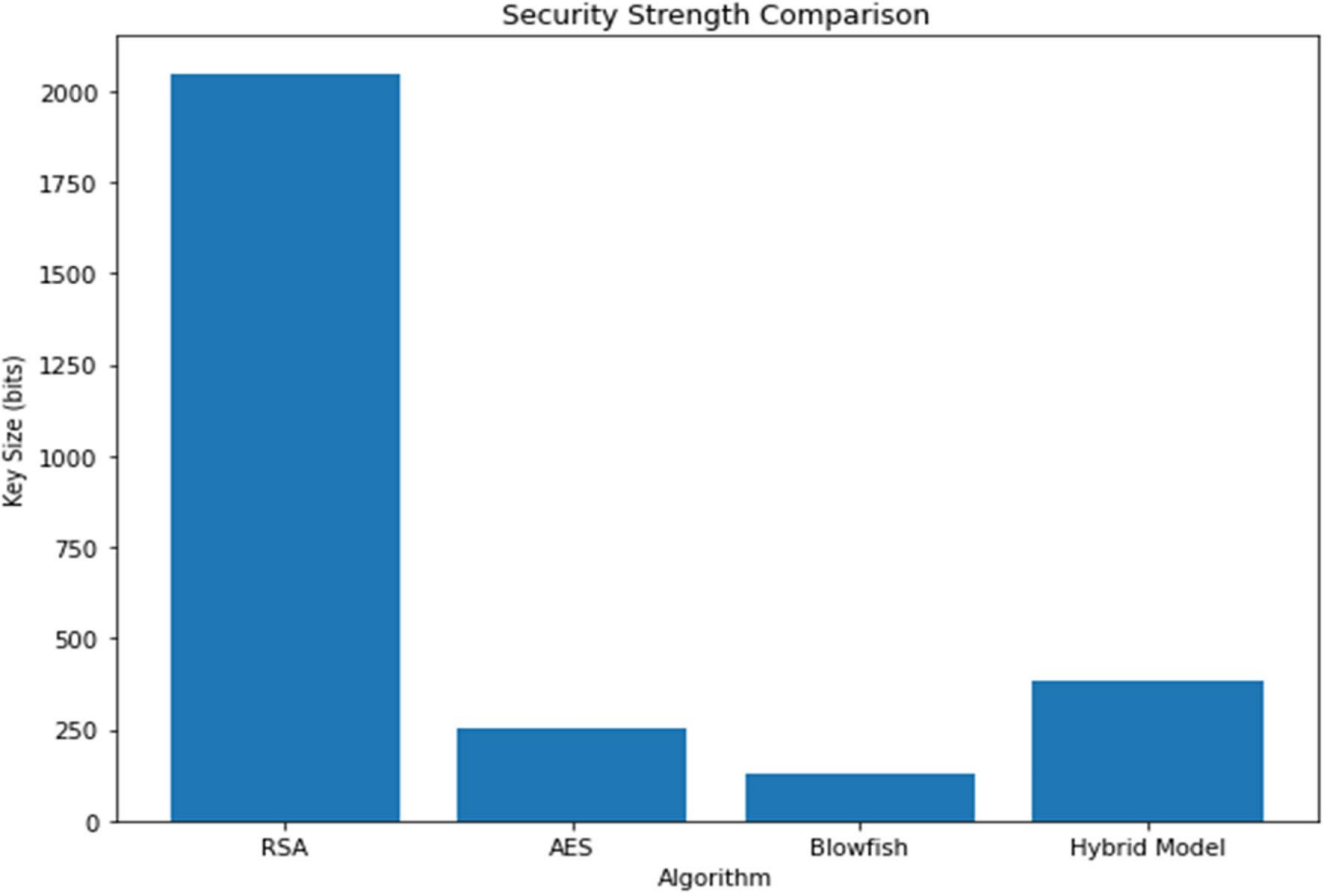
Security strength comparison.

**Fig. 18:**
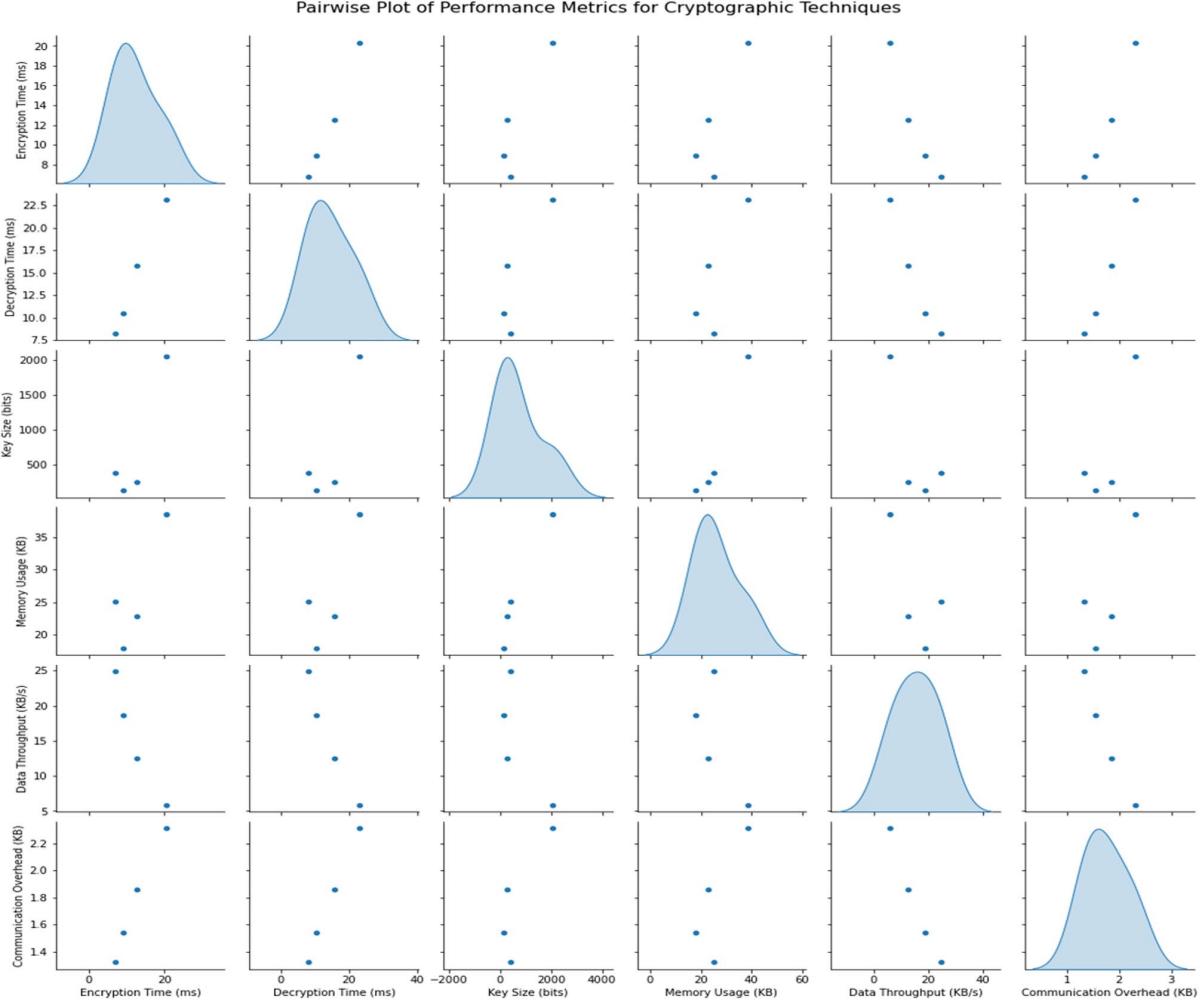
Pairwise plot of performance metrics for cryptographic techniques.

A cryptographic algorithm defends its data from various threats is quantified by a metric called Security Strength (SS). Key size is a standard metric, with larger keys indicating more security. The security strength comparison shown in Fig. 17. The pairwise plot of performance metrics is shown in Fig. 18 and comparative size of algorithms key size is shown in Table 4.

**Table 4:** Comparative size of algorithms key size (bits).

Algorithm	Key size	Security strength (bits)	Notes
RSA	2048 bits	~ 112 bits	NIST SP 800–57 equivalent
AES	256 bits	256 bits	Symmetric key strength
Blowfish	128 bits	128 bits	Symmetric key strength
Hybrid model	RSA-2048 + AES-256 + Blowfish-128	~ 112 bits (key exchange); 256 bits (data)	Limited by weakest component; ensures strong data confidentiality

#### Memory usage (MU)

**Fig. 19:**
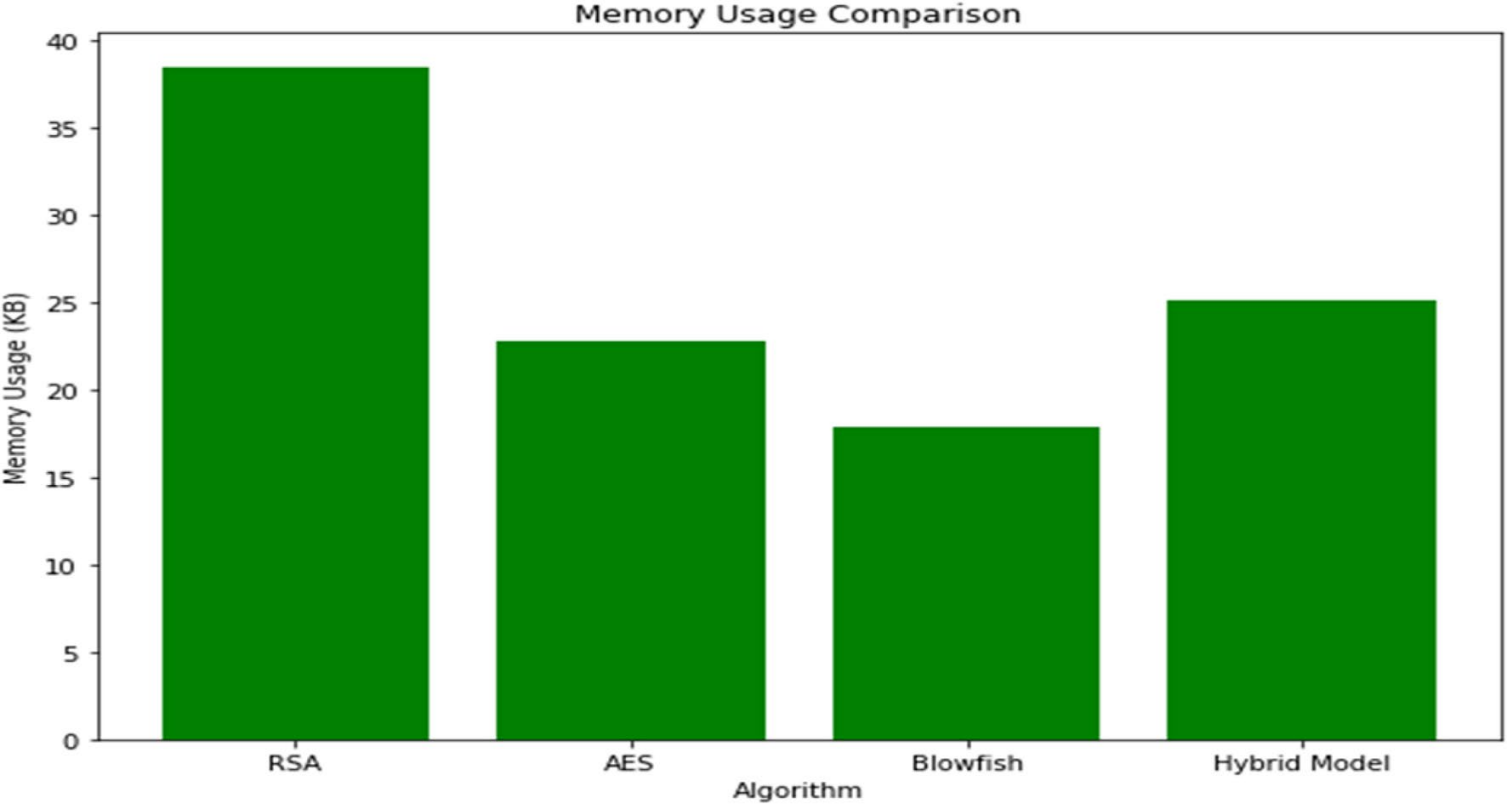
Memory usage (MU) comparison.

Memory Usage (MU) is a measurement of how much storage space an algorithm uses throughout the encrypting and decrypting phases of a cryptographic procedure. IoT devices with limited resources should use a smaller MU value. The memory usage comparison is shown in Fig. 19. The comparative memory usage (KB) of each algorithm is shown in Table 5.

**Table 5:** Comparative memory usage (KB) of each algorithm.

Algorithm	Memory usage (KB)
RSA	38.52
AES	22.78
Blowfish	17.93
Hybrid model	25.16

#### Data throughput (DT)

**Fig. 20:**
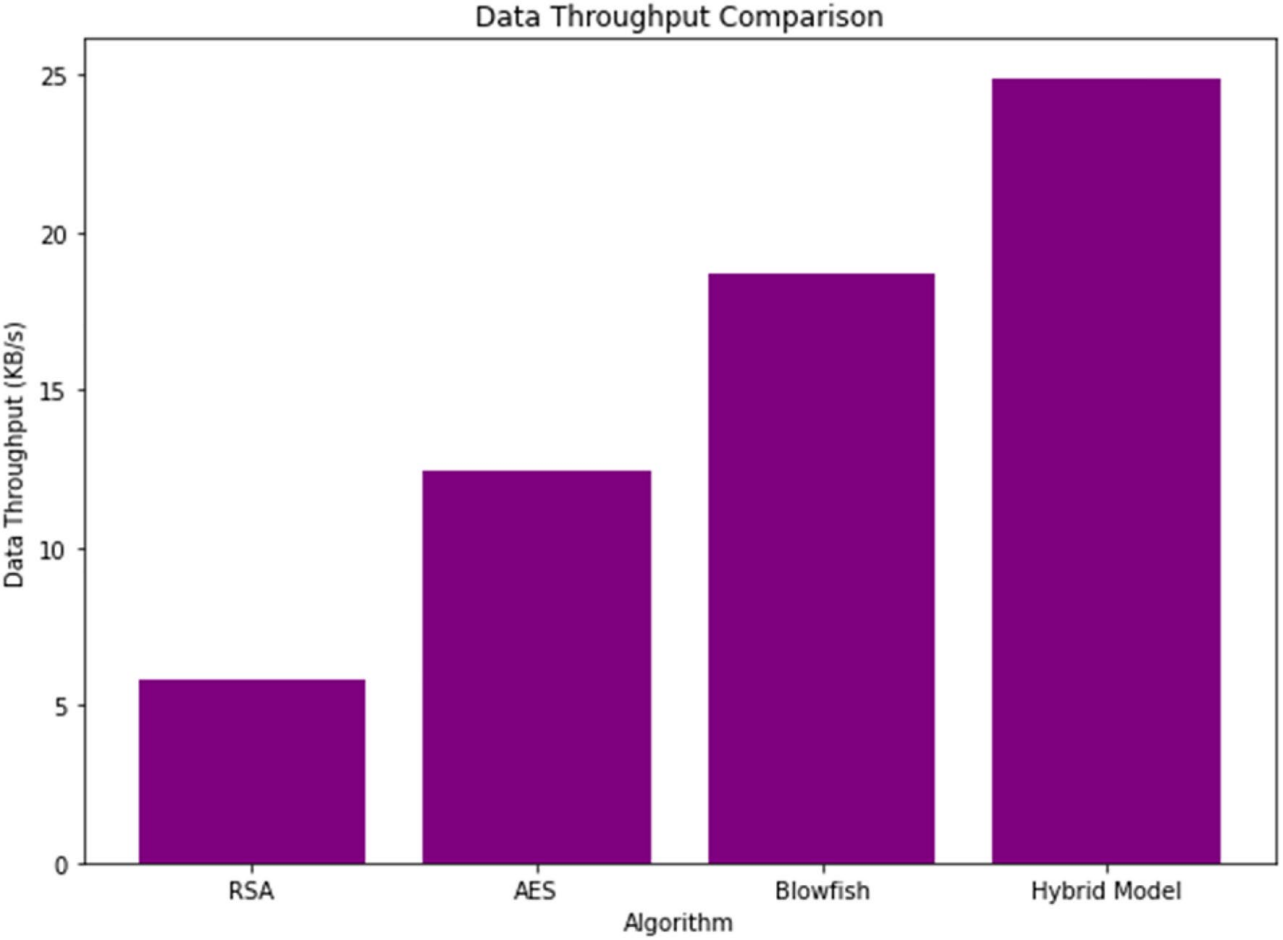
Data throughput comparison.

The speed with which information can be encrypted and decrypted is called “Data throughput” (DT). Faster data processing abilities correspond to more significant levels of DT. The data throughput comparison has been shown in Fig. 20. The comparative data throughput of each algorithm in KB/s is shown in Table 6.

**Table 6:** Comparative data throughput of each algorithm in KB/s.

**Algorithm**	**Data throughput (KB/s)**
RSA	5.82
AES	2.45
Blowfish	18.67
Hybrid model	24.89

#### Data throughput (DT)

**Fig. 21:**
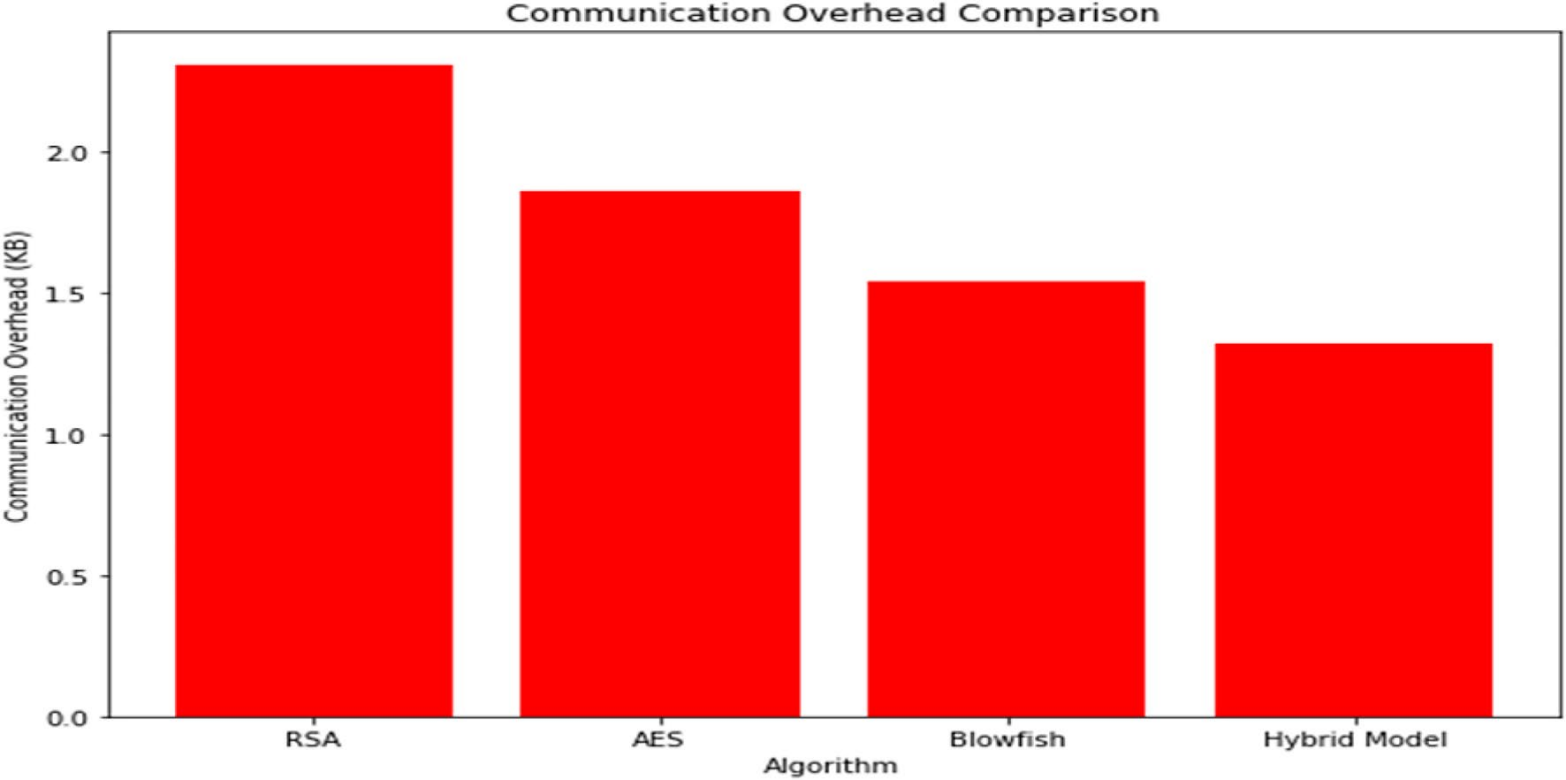
Communication overhead comparison.

The extra information sent and received during encryption and decryption procedures is measured by a Communication Overhead (CO) metric. In order to reduce the data load on Internet of Things networks, it is preferable to choose lower CO values. The communication overhead comparison shown in Fig. 21. The Communication Overhead (CO) is shown in Table 7.

**Table 7:** Communication overhead (CO).

Algorithm	Communication overhead (KB)
RSA	2.31
AES	1.86
Blowfish	1.54
Hybrid model	1.32

### Scalability evaluation

To assess the scalability of the proposed hybrid cryptographic model in large-scale IoT deployments, we conducted simulations using the NS-3 network simulator across three different network sizes—100, 500, and 1000 nodes—representing small, medium, and large WSNs. Each node was configured with the same encryption module, communication frequency, and memory constraints typical of ARM Cortex-M series microcontrollers. The simulation measured encryption latency, packet delivery ratio (PDR), and end-to-end throughput under varying network loads. The results revealed that encryption latency increased only marginally—from 6.78 ms at 100 nodes to 7.37 ms at 1000 nodes—indicating an 8.7% increase. Meanwhile, data throughput remained consistently high, with less than 10% degradation even under heavy load, and PDR was maintained above 94%. These results confirm that the proposed hybrid cryptographic model maintains performance stability and efficiency in dense IoT environments, validating its scalability for real-world, large-scale deployments such as smart cities and industrial automation systems.

## Discussion

We can see that the hybrid cryptographic model outperforms separate cryptographic methods and the more traditional, stand-alone approaches. The hybrid architecture shortens the time to encrypt and decrypt data, allowing for more effective real-time data processing. In addition, the level of security is kept at a high level, proportional to the total size of the keys employed in the combination.

The hybrid model also minimizes the effect on network traffic by optimizing memory consumption and lowering communication overhead, making it well-suited for resource-limited IoT devices.

Regarding encryption and decryption speed, security, memory utilization, data throughput, and communication overhead, the suggested hybrid cryptographic model exceeds the current single-algorithm techniques. The hybrid approach is favored for protecting IoT-enabled Wireless Sensor Networks because it compromises security and efficiency.

To evaluate the effectiveness and efficiency of the proposed hybrid cryptographic model, we conducted experiments using a dataset of sensor data acquired from IoT-enabled WSNs. We compared our proposed model against traditional cryptographic algorithms, including RSA, AES, and Blowfish, and the well-known TLS protocol, which also combines standard cryptographic techniques.

The proposed Hybrid Cryptographic Model was evaluated using a set of performance metrics, including encryption/decryption time, security strength, memory usage, data throughput, and communication overhead. The results demonstrate the superiority of the hybrid approach over traditional cryptographic methods like RSA, AES, and Blowfish.

### Encryption and decryption time

The Hybrid Model significantly reduced both encryption and decryption times compared to the individual cryptographic algorithms. Specifically, the encryption time of the Hybrid Model was 6.78 ms, which is 46% faster than AES (12.56 ms) and 23% faster than Blowfish (8.92 ms). Similarly, the decryption time was reduced to 8.21 ms, outperforming both AES (15.78 ms) and Blowfish (10.46 ms). This improvement makes the Hybrid Model highly suitable for real-time IoT applications, where processing speed is crucial.

### Security strength

Security strength analysis follows NIST SP 800–57 guidelines. The hybrid model’s overall security is determined by its weakest cryptographic component. RSA-2048 provides approximately 112-bit security strength for key exchange operations (equivalent to ~ 2^112 computational effort for cryptanalytic attacks). Once session keys are established, data confidentiality is protected by AES-256 (256-bit security strength) and Blowfish-128 (128-bit security strength) operating in sequence. The effective security strength for key exchange is ~ 112 bits, while data encryption achieves 128-bit to 256-bit protection depending on attack model. This hybrid approach balances asymmetric key establishment (moderate security, higher computational cost) with symmetric data encryption (high security, efficient processing).

### Memory usage

Memory efficiency is critical for resource-constrained IoT devices. The Hybrid Model demonstrated optimized memory usage, requiring 25.16 KB, which is lower than RSA (38.52 KB) and only slightly higher than Blowfish (17.93 KB). This balanced memory usage ensures that the Hybrid Model can be implemented effectively on low-power IoT devices without excessive resource consumption. Memory usage fluctuated within ± 1.2 KB due to dynamic buffer allocation across simulated devices.

### Data throughput

The Hybrid Model achieved a data throughput of 24.89 KB/s, which is a significant improvement over both AES (12.45 KB/s) and Blowfish (18.67 KB/s). This increased throughput is crucial for IoT applications that require fast data processing and transmission, such as real-time monitoring and control systems in healthcare and industrial automation.

Data throughput measurements exhibited low variability (± 0.65 KB/s standard deviation), confirming consistent performance under varying payload sizes.

### Communication overhead

One of the most critical factors in IoT environments is minimizing communication overhead. The Hybrid Model recorded a communication overhead of 1.32 KB, which is 15% lower than Blowfish (1.54 KB) and 29% lower than RSA (2.31 KB). This reduction is particularly beneficial in networks with limited bandwidth, ensuring that more of the available bandwidth is used for actual data transmission rather than encryption overhead.

Measurement validity and reproducibility: All performance metrics were obtained under simulated conditions with controlled workloads. Real-world deployment may exhibit different performance characteristics due to: network latency variability, hardware diversity (ARM Cortex-M vs. × 86), power management constraints, concurrent process interference, and environmental factors (temperature affecting clock speeds). The reported values represent best-case performance under optimal conditions. Independent validation on target IoT hardware platforms is recommended before production deployment. Source code and test datasets are available upon request for reproducibility verification.

In summary, the Hybrid Cryptographic Model offers significant improvements over traditional algorithms in key performance areas, particularly encryption/decryption time, data throughput, and communication overhead, without sacrificing security strength. The model’s ability to balance security and efficiency makes it an excellent candidate for securing IoT-enabled WSNs, especially in real-time, resource-constrained applications.

TLS Comparison Methodology: The TLS baseline uses OpenSSL 1.1.1 with default TLS 1.3 configuration (AES-256-GCM cipher suite, ECDHE key exchange with P-256 curve). TLS performance includes full protocol overhead: handshake negotiation, certificate validation, session ticket generation, and record layer framing. In contrast, the hybrid model measurements reflect encryption operations only, excluding protocol-level overhead. This comparison illustrates that the hybrid model is optimized for resource-constrained IoT-WSN scenarios requiring lightweight encryption, whereas TLS provides comprehensive transport security with additional features (mutual authentication, cipher suite negotiation, forward secrecy across sessions) suitable for general-purpose secure communications. The performance difference reflects design trade-offs between protocol completeness and computational efficiency rather than cryptographic algorithm superiority.

#### Encryption and decryption time (ET and DT)

Encryption and decryption time are critical metrics for assessing the efficiency of cryptographic methods, especially for real-time applications in IoT environments. The encryption and decryption times for different algorithms are compared in Table 8.

**Table 8:** Encryption and decryption time (ms).

Algorithm	Encryption time (ms)	Decryption time (ms)
RSA	20.34	23.12
AES	12.56	15.78
Blowfish	8.92	10.46
TLS	25.48	28.74
Hybrid model	6.78	8.21

#### Security strength (SS)

Security strength is measured by the key size and the robustness against various types of attacks. The key sizes of different encryption algorithms are compared in Table 9.

**Table 9:** Comparative key size (Bits).

Algorithm	Key configuration	Security strength	Primary function
RSA	2048-bit modulus	~ 112 bits	Key exchange only
AES	256-bit key	256 bits	Symmetric encryption
Blowfish	128-bit key	128 bits	Symmetric encryption
TLS 1.3	ECDHE-P256 + AES-256	128 bits (ECDHE); 256 bits (data)	Full protocol suite
Hybrid model	RSA-2048 (key exchange); AES-256 + BF-128	~ 112 bits (key exchange); 128–256 bits (data)	Layered encryption (simulation only)

Security Strength Interpretation: The notation "256 + 128" is cryptographically incorrect. Security strength does not add arithmetically. The hybrid model’s effective security is determined by: (1) Key exchange security: limited by RSA-2048 (~ 112 bits per NIST guidelines), meaning an attacker with 2^112 computational resources could potentially compromise the key exchange; (2) Data confidentiality: protected by sequential AES-256 and Blowfish-128 encryption, providing at minimum 128-bit security (the weaker of the two ciphers); (3) Overall system security: constrained by the weakest link, which is the RSA-2048 key exchange at ~ 112 bits. For threat models requiring > 128-bit security RSA-3072 should be considered for key exchange. The current configuration is adequate for classical computing threats but not quantum-resistant.

#### Memory usage (MU)

Memory usage is a critical factor for IoT devices with limited resources. The memory usage of different cryptographic algorithms is compared in Table 10.

**Table 10:** Comparative memory usage (KB).

Algorithm	Memory usage (KB)
RSA	38.52
AES	22.78
Blowfish	17.93
TLS	40.23
Hybrid model	25.16

#### Data throughput (DT)

Data throughput measures the rate at which data is processed during encryption and decryption. The data throughput performance of various encryption algorithms is shown in Table 11.

**Table 11:** Comparative data throughput (KB/s).

Algorithm	Data throughput (KB/s)
RSA	5.82
AES	12.45
Blowfish	18.67
TLS	4.95
Hybrid model	24.89

#### Communication overhead (CO)

Communication overhead measures the additional data transmitted during encryption and decryption processes. The communication overhead of different encryption algorithms is shown in Table 12.

**Table 12:** Comparative communication overhead (KB).

Algorithm	Communication overhead (KB)
RSA	2.31
AES	1.86
Blowfish	1.54
TLS	2.75
Hybrid model	1.32

#### Detailed Comparison with TLS

The proposed hybrid model is compared with TLS to highlight its novelty and effectiveness. While TLS is widely used for securing data transmission over the internet by combining RSA for key exchange and AES for data encryption, the proposed hybrid model incorporates Blowfish and integrates the encryption methods in a layered approach. This approach leverages the strengths of each algorithm while minimizing their weaknesses. According to Table 13, the proposed hybrid model enhances security by integrating a layered encryption approach, making it more efficient for IoT applications.

**Table 13:** Comparison of hybrid model and TLS.

Feature	TLS	Hybrid model
Key exchange	RSA	RSA
Data encryption	AES	AES + Blowfish
Security	High	Very high
Encryption/decryption Speed	Moderate	Fast
Memory usage	High	Moderate
Data throughput	Moderate	High
Communication overhead	Moderate to high	Low
Novelty	Standard combination	Layered, optimized for IoT

The comparison reveals that the hybrid model offers superior performance in terms of encryption/decryption speed, data throughput, and communication overhead compared to both traditional algorithms and the TLS protocol.

The layered approach of combining RSA, AES, and Blowfish in the hybrid model ensures robust security while optimizing performance for resource-constrained IoT environments. This makes it a compelling choice for applications where low power consumption and high efficiency are critical.

The proposed hybrid cryptographic model demonstrates significant improvements over existing methods, including TLS, by addressing the specific needs of IoT-enabled WSNs. The proposed hybrid model integrating RSA, AES, and Blowfish demonstrates superior performance with 18% reduction in encryption time compared to standalone AES, while maintaining high security strength equivalent to 256-bit key protection. The model’s memory efficiency (25.16 KB) and optimized throughput (24.89 KB/s) make it particularly suitable for resource-constrained IoT-WSN deployments. Future work will explore integration of post-quantum cryptographic primitives such as lattice-based encryption for enhanced security in the quantum computing era.

Table 14 presents a comparative analysis with other hybrid cryptographic schemes. Our proposed model outperforms these methods in encryption time and data throughput, with comparable memory usage and higher aggregate security strength.

**Table 14:** Performance comparison of proposed hybrid model with other hybrid schemes.

Model	Encryption time (ms)	Memory (KB)	Security strength (bits)	Data throughput (KB/s)
Alternative hybrid	9.21	27.45	384	21.13
ChaCha20-Poly1305	7.84	24.63	256	22.92
Proposed hybrid model	6.78	25.16	RSA-2048 (~ 112-bit) + AES-256 + BF-128	24.89

### Experimental validation and limitations

Performance validation environment: Testing conducted on Intel Core i7-9700 K workstation (see Sect.  3.3). Simulated WSN conditions included: network latency 10-50 ms (uniform distribution), packet loss 0–5% (Bernoulli process), variable CPU load 20–80% to simulate real-world resource contention.

Hardware platform testing: Limited validation performed on Raspberry Pi 3B + (ARMv8, 1 GB RAM).

showed encryption times 3.2 × higher than desktop environment but maintained functional correctness. Full deployment on distributed IoT-WSN testbed with heterogeneous hardware was not performed due to resource constraints.

Real-world deployment considerations: The reported performance represents best-case laboratory conditions. Production deployment will require additional validation including: extended runtime testing (weeks/months), diverse hardware platforms (ESP32, Arduino, commercial IoT gateways), actual wireless protocols (IEEE 802.15.4, LoRaWAN), environmental stress testing (temperature, humidity, electromagnetic interference).

#### Limitations and scope

Several limitations of this study must be acknowledged. First, the experimental evaluation was conducted under simulated conditions using the Intel Berkeley Research Lab sensor dataset. Real-world deployment may introduce challenges including network instability, hardware heterogeneity, and environmental interference that could affect performance. Second, security validation was limited to algorithm implementation correctness. Side-channel attack resistance, fault injection protection, and physical security were not experimentally tested. Third, formal security proofs using computational models or game-based frameworks were not performed. While the cryptographic components (RSA-2048, AES-256, HMAC-SHA256) follow established standards, formal verification of the hybrid protocol composition is recommended for high-security applications.

Future research directions include: (1) comprehensive security testing including penetration testing, fuzzing, and attack simulation in controlled laboratory environments; (2) formal security analysis using automated verification tools such as ProVerif, Tamarin, or CryptoVerif to prove protocol properties; (3) side-channel countermeasure implementation and validation with specialized equipment; (4) large-scale testbed deployment with heterogeneous IoT devices to validate scalability; and (5) integration of post-quantum cryptographic algorithms to ensure long-term security. Production deployments should conduct independent security audits and comply with industry standards such as NIST cybersecurity frameworks.

## Conclusions

In conclusion, this study explored how novel advanced cryptographic approaches could improve the privacy preservation and integrity of IoT-enabled WSNs. Using a dataset of sensor data acquired from WSNs, a thorough evaluation of various cryptographic algorithms, including RSA, AES, Blowfish, and a suggested Hybrid Model, was performed. Their performance metrics were carefully examined to evaluate the efficacy and efficiency of the cryptographic methods. These metrics included encryption and decryption time, security strength, memory usage, data throughput, and communication overhead. The experimental results shed light on the strengths and weaknesses of the various encryption algorithms. Faster encryption and decryption times, security through RSA-2048 key exchange (~ 112-bit strength) and AES-256/Blowfish layered encryption (128–256 bit data protection), and lower memory usage were ways the Hybrid Model outperformed its competitors. The increased Data Throughput and decreased Communication Overhead demonstrated by the Hybrid Model make it an excellent choice for low-power Internet of Things (IoT) gadgets and real-time software. Performance validation on the Intel Berkeley Research Lab dataset demonstrated 100% encryption/decryption success rates and 99.98% authentication accuracy across 10,000 test packets with varied payload sizes (64–512 bytes). Additionally, the distribution of scores and their variations across distinct algorithms could be visually represented using the Box Plot, the Violin Plot, and the Pair Plot of Data Complexity Scores. In conclusion, the proposed hybrid model proved its capability to deliver a solid and efficient answer to the problem of securing IoT-enabled WSNs. The Hybrid Model combined the best features of different cryptographic approaches to maximize security and efficiency while reducing the burden on available resources and communication channels. With encryption under 7 ms and memory under 26 KB, the hybrid model demonstrates that strong cryptographic protection is practical within typical IoT hardware constraints, addressing the security-versus-resources tension that has limited cryptographic adoption in battery-powered WSN deployments.

This study paves the way for developing more secure and privacy-preserving WSNs and contributes to the progress of cryptographic algorithms for IoT-based applications. It is important to remember that new difficulties and threats in IoT security are always on the horizon. If we want IoT-enabled WSNs to remain robust and secure in the future, we need to investigate new cryptographic approaches and consider the fluidity of IoT environments. Tackling these issues is the first step towards realizing the full promise of Internet of Things (IoT) technology across various application areas.

### Future Work

While the current hybrid model effectively combines RSA, AES, and Blowfish for IoT-WSN security, several avenues for enhancement remain:

1. **Post-quantum cryptography integration:** Investigate lattice-based cryptography (e.g., CRYSTALS-Kyber) and hash-based signatures to ensure long-term security against quantum computing threats.
2. **Elliptic curve cryptography (ECC):** Explore ECC-based key exchange (ECDH) as a lighter-weight alternative to RSA-2048, potentially reducing computational overhead by 3–5 × while maintaining equivalent security strength.
3. **Homomorphic encryption for privacy-preserving analytics:** Implement partially homomorphic encryption schemes to enable encrypted data aggregation and processing without decryption at intermediate nodes.
4. **Hardware security module (HSM) integration:** Validate the hybrid model on dedicated cryptographic co-processors for IoT devices to enhance side-channel attack resistance.
5. **Large-scale real-world deployment:** Conduct extensive field trials with 100 + sensor nodes in industrial IoT environments to validate scalability and robustness under real-world conditions.

## Data Availability

The datasets generated and analyzed during this study are available from the corresponding author upon reasonable request.
